# YTHDC1 Orchestrates Telomerase Assembly via Scaffold‐Mediated TERT‐*TERC* Interaction

**DOI:** 10.1111/acel.70332

**Published:** 2025-12-28

**Authors:** Xiaolei Cheng, Shixing Wang, Yanan Yu, Jianhang Xu, Qian Wang, Yuzhu Wei, Zeming Jin, Xinkun Qi, Dongdong Jian, Yingchao Shi, Zhen Li, Zhengliang Ma, Wengong Wang, Tianjiao Xia, Junyue Xing, Xiaoping Gu, Hao Tang

**Affiliations:** ^1^ Zhengzhou Key Laboratory of Cardiovascular Aging, National Health Commission key Laboratory of Cardiovascular Regenerative Medicine Central China Fuwai Hospital of Zhengzhou University, Fuwai Central China Cardiovascular Hospital & Central China Branch of National Center for Cardiovascular Diseases Zhengzhou Henan China; ^2^ Department of Anesthesiology Affiliated Drum Tower Hospital of Medical School of Nanjing University Nanjing Jiangsu China; ^3^ Department of Biochemistry and Molecular Biology, Beijing Key Laboratory of Protein Posttranslational Modifications and Cell Function, School of Basic Medical Sciences Peking University Health Science Center Beijing China; ^4^ School of Life Sciences and Technology Henan Medical University, Institute of Cardiovascular Disease, Henan Academy of Innovations in Medical Science Zhengzhou Henan China; ^5^ Medical School Nanjing University, Jiangsu Key Laboratory of Molecular Medicine, Nanjing University Nanjing China

**Keywords:** RNA methylation, telomerase assembly, telomere associated disease, YTHDC1

## Abstract

Telomerase RNA (*TERC*) is subject to various modifications, yet the implications of these modifications for telomerase biology remain largely unexplored. In this study, we conducted a comprehensive mapping of N6‐Methyladenosine (m6A) modifications within *TERC* RNA and elucidated their regulatory role in telomerase function. Our findings demonstrate that *TERC* undergoes methylation at adenosine residues A111 and A435 by METTL3. A deficiency in *TERC* m6A, which is also linked to various human telomerase disease‐related mutations and deletions, significantly reduces telomerase activity and telomere length by disrupting the association between *TERC* and TERT. Mechanistically, YTHDC1 was identified as a scaffold facilitating the interaction between TERT and *TERC,* binding to TERT while recognizing m6A sites on *TERC*. Knockdown of YTHDC1 significantly diminished the interaction between TERT and *TERC*, thereby reducing telomerase activity and phenocopying the deficiency of METTL3. Furthermore, reconstituting wild‐type YTHDC1 rescued telomere attrition, proliferation defects, and senescence in YTHDC1‐knockdown alveolar epithelial cells, whereas truncated YTHDC1 (which retains m6A recognition but lacks TERT‐binding capacity) failed to restore these phenotypes. Collectively, our work establishes m6A modification of *TERC* as a central regulator of telomerase function and reveals YTHDC1's scaffolding role in TERT‐*TERC* assembly, shedding new light on the regulation of telomerase and related diseases.

## Graphical abstract

Schematic illustration of the major findings: METTL3‐deposited m6A modifications on *TERC* recruit the reader protein YTHDC1, which functions as a molecular scaffold to facilitate telomerase holoenzyme assembly by bridging TERT to m6A‐modified *TERC* transcripts, thereby promoting the proliferation of AECs.

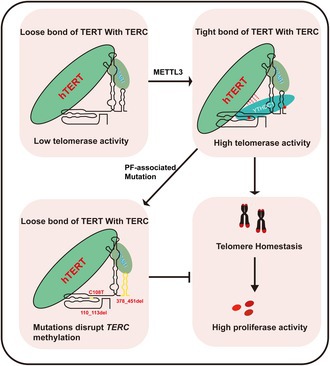

## 1 Introduction

Telomeres, composed of tandem repeat DNA sequences of TTAGGG and telomeric‐associated proteins, are essential structural components of eukaryotic linear chromosomes that safeguard genome stability (Wang et al. 2018; Fu and Collins 2007; Lundblad and Szostak 1989). Critically shortened telomeres trigger end‐to‐end chromosome fusion and cellular senescence, thereby limiting proliferative capacity (Chakravarti et al. 2021; Blackburn et al. 2015; Opresko and Shay 2017). Telomerase, a ribonucleoprotein complex comprising the catalytic subunit TERT and the template RNA *TERC*, counteracts telomere attrition by elongating chromosome ends with TTAGGG repeats, thereby preserving genomic integrity (Blackburn et al. 2015; Schmidt and Cech 2015). Although the expression of TERT and *TERC* is fundamental, the in vivo assembly of the functional telomerase holoenzyme is contingent upon multiple auxiliary factors. These regulatory components are critical for fine‐tuning telomerase activity, a process that markedly differs from in vitro reconstitution (Venteicher et al. 2009; Masutomi et al. 2000; Deng et al. 2019). A comprehensive understanding of these accessory components would significantly advance our insight into the mechanisms that control telomerase assembly and regulation.

Previous studies have demonstrated that cellular *TERC* levels exceed those incorporated into active telomerase (Liu et al. 2019; Blasco et al. 1996). Consequently, the processing, subcellular localization, and structural integrity of *TERC* are posited to be crucial for the assembly and function of telomerase. Indeed, multiple factors such as genetic variations, processing enzymes, and RNA‐binding proteins, which are proposed to influence the post‐transcriptional maturation events of *TERC*, have been extensively characterized in telomere‐related diseases (TADs), including pulmonary fibrosis, dyskeratosis congenita, and aplastic anemia, in recent years (Mitchell et al. 1999; O'Connor et al. 2006; Xin et al. 2007; Chen et al. 2018; Tang et al. 2018; Xu et al. 2020; Markiewicz‐Potoczny et al. 2021). For instance, *TERC* genetic variants (C108T, 110_113del, 378_451del) have been reported to be significantly linked to pulmonary fibrosis susceptibility (Podlevsky et al. 2008). Furthermore, the RNA‐binding protein ELAVL1 is suggested to enhance telomerase assembly and activity by maintaining *TERC* RNA cytosine methylation, a process disrupted by genetic mutations within *TERC* closely associated with dyskeratosis congenita (Cheng et al. 2021; Tang et al. 2018). These findings underscore that functional telomerase production depends on tightly regulated *TERC* maturation, a process requiring further mechanistic investigation.

RNA modifications constitute a crucial aspect of post‐transcriptional regulation, dynamically governed by ‘writer’ (depositing) and ‘eraser’ (removing) proteins. These modifications primarily influence RNA metabolism and function by recruiting specific reader proteins (e.g., YTHDF, YTHDC, IGF2BP, and YBX families) and/or modulating RNA structure (Fu et al. 2014; Zaccara et al. 2019; Boulias and Greer 2023). To date, only a few modifications have been identified within *TERC* RNA, including N7‐methylguanosine (m7G), 5‐methylcytosine (m5C), and pseudouridine (Ψ) (Tang et al. 2018; Squires et al. 2012; Zemora et al. 2016; Chen et al. 2020). Recent advancements in MS2‐ and Cas13‐based apex‐targeting techniques have confirmed the interaction between *TERC* and the N6‐Methyladenosine (m6A) demethylase ALKBH5 (Han et al. 2020), suggesting a potential regulatory role for m6A dynamics in *TERC*. However, the functional significance and mechanistic basis of the m6A modification of *TERC* remain to be elucidated.

In this study, we systematically investigated the m6A modification of *TERC*, revealing that METTL3‐mediated methylation predominantly occurs at adenosine residues A111 and A435. Notably, pulmonary fibrosis (PF)‐associated mutations (C108T, 110_113del, and 378_451del) substantially diminished *TERC* m6A methylation. A deficiency in *TERC* m6A severely impaired telomerase activity and exacerbated telomere shortening. Mechanistically, the m6A reader YTHDC1 was found to selectively recognize the methylated sites and directly bound TERT, functioning as a molecular scaffold that bridges TERT and methylated *TERC* to facilitate telomerase holoenzyme assembly. Importantly, reintroduction of wild‐type YTHDC1, but not a truncated variant lacking the TERT‐binding domain, restored proliferation capacity and mitigated senescence in YTHDC1‐deficient alveolar epithelial cells. Our findings unveil a novel regulatory mechanism by which RNA methylation governs telomerase activity and highlight the dual architectural role of YTHDC1 as both an m6A reader and a molecular scaffold important for TERT‐*TERC* holoenzyme assembly.

## 2 Methods

### 2.1 Cell Culture and Transfection

Human alveolar epithelial cells (AECs) were purchased from ATCC and cultured at 37°C in 5% CO_2_ with Dulbecco's modified Eagle's medium (DMEM, Gibco, USA) supplemented with 10% fetal bovine serum and 100 units/mL penicillin and streptomycin. Cells were transfected with 100 pmol METTL3 siRNA (siMETTL3), YTHDC1 siRNA (siYTHDC1), or negative control siRNA (siNC) using Lipofectamine RNAiMAX transfection reagent (Invitrogen, USA), according to the manufacturer's instructions. The corresponding siRNA sequences are listed in Table S1. Plasmid transfections were performed using Lipofectamine 3000 (Invitrogen) according to standard protocols. To construct stable knockdown cell lines, viruses expressing shCtrl, shMETTL3, or shYTHDC1 shRNA were packaged using a triple plasmid system (psPAX2 and pMD2.G) and transfected into AECs. Positive cells were screened using puromycin (2 ug/mL) to establish stable polyclonal populations.

### 2.2 Plasmid Construction

To construct PHBLV‐shMETTL3‐GFP or PHBLV‐ shYTHDC1‐GFP plasmids, oligos of shRNA listed in Table S1 were purchased and annealed to double‐stranded shRNA, subsequently inserted between BamH I and EcoR I sites of the PHBLV‐U6‐GFP plasmid.

### 2.3 Construction of the m6A‐Editing System

For targeted m6A demethylation, AECs were co‐transfected with *TERC* targeting sgRNAs (*TERC*‐sgRNA‐1 and sgRNA‐2, listed in Table S1) and the dCas13‐ALKBH5 plasmid using Lipofectamine3000. LacZ‐sgRNA was used as a negative control. Transfected cells were harvested 48 h post‐transfection for downstream analyses.

### 2.4 Western Blotting and Real Time Quantitative PCR (Q‐PCR)

Western blotting was performed according to standard procedures. Antibodies listed in Table S1 were used to detect the respective bands at 4°C overnight.

Total RNA was extracted from cells using the Trizol reagent (Invitrogen) following the manufacturer's protocol. After reverse transcription, cDNA was subjected to real‐time quantitative PCR (Q‐PCR) to detect the expression of the target gene. The primer pairs used in this study are provided in Table S1.

### 2.5 RNAscope Fluorescent Assay

The RNAscope Multiplex Fluorescent Assay (ACD Bio, USA) was performed according to the manufacturer's protocol. Briefly, AECs were initially seeded into confocal Petri dishes and fixed with 10% neutral formalin. Subsequently, the cells were subjected to digestion with RNAscope Protease III for 10 min, and the digestion was terminated by washing with PBS. This was followed by probe hybridization, signal cascade amplification with a Z probe and multi‐channel fluorescent staining to label the endogenous *TERC* RNA. The cells were then blocked with 5% BSA for 1 h at room temperature. Subsequently, the cells were incubated with a primary antibody against YTHDC1 overnight at 4°C. After washing three times with PBST (containing 0.1% Tween 20), a fluorescent secondary antibody (1:200) with the corresponding properties was added and incubated for 2 h at room temperature. The cells were washed again with PBST, followed by counterstaining with a blocking agent containing 4′,6‐diamidino‐2‐phenylindole (DAPI) to visualize nuclei. The co‐localization of YTHDC1 and *TERC* RNA within the cells was then visualized and imaged using confocal fluorescence microscopy.

### 2.6 Telomere Repeat Amplification Protocol (TRAP), immunoprecipitation TRAP (IP‐TRAP), and Telomere‐Restricted Fragment Length Assay (TRF)

TRAP and IP‐TRAP assays were performed as previously described (Tang et al. 2018). Briefly, the cells were collected and lysed using NP‐40 lysis buffer (150 mM NaCl, 10 mM Tris–HCl pH 8.0, 1 mM MgCl_2_, 1 mM EDTA, 1% NP‐40, 0.25 mM Sodium deoxycholate, 5 mM β‐ME, and protein inhibitor) at a concentration of 1000 cells/μL. Two microliters of lysate were subjected to the extension reaction at 37°C incubation and followed by PCR amplification with TS, ACX, NT, and TSNT primers. The sequences are listed in Table S1. The products were separated on non‐denatured polyacrylamide gels (12%) and stained with SYBR Safe (Invitrogen). The bands were visualized using an Odyssey system. The intensity of the bands was quantified using image J and the intensity of the telomere bands was calculated against that of the internal control. For IP‐TRAP, the cells were lysed in lysis buffer (0.01% NP‐40,10 mM Tris pH 7.5, 50 mM KCl, 5 mM MgCl_2_, 2 mM dithiothreitol, 20% glycerol, and protease inhibitors) at a concentration of 100,000 cells/mL and sonicated at 50 J/W‐s. After centrifugation at 16,000 × *g* for 30 min, the supernatant was collected and used for immunoprecipitation with the corresponding antibody at 4°C for 4–6 h. The mixture was then added to protein A/G agarose beads (Invitrogen) and incubated at 4°C for 1 h. After washing with lysis buffer four times, the beads were resuspended in lysis buffer and subjected to a TRAP assay to detect telomerase activity.

A telomere‐restricted fragment length assay was performed using a commercial kit (cat. no. 12 209136 001; Roche, Switzerland) according to the manufacturer's instructions. Briefly, genomic DNA was digested using HinfI and RsaI and then separated using a 0.8% agarose gel. After fixing, denaturing, and neutralizing, the gel was transferred to a nylon membrane. Subsequently, the membrane was UV‐crosslinked, hybridized with a Digoxin‐labeled TTAGGG probe, and incubated with an alkaline phosphatase‐coupled anti‐digoxin antibody. After addition of the reaction substrate, the membrane was exposed to a chemiluminescence apparatus (Thermo Fisher, USA). Mean TRF length has been defined according to the following formula:

$$\bar{\mathrm{TRF}}=\frac{\sum \left({\mathrm{OD}}_{i}\right)}{\sum \left({\mathrm{OD}}_{i}/L_{i}\right)}$$

where OD_*i*_ is the chemiluminescent signal and *L*_*i*_ is the length of the TRF at position *i*. The calculation considered the higher signal intensity from larger TRFs due to multiple hybridizations of the telomere‐specific hybridization probe.

### 2.7 RNA Pulldown and Immunoprecipitation

RNA pull‐down experiments were performed as previously described (Liu et al. 2022) with the following modifications. Biotinylated *TERC* RNA probes (wild‐type and mutant variants) were synthesized in vitro using the TranscriptAid T7 High‐Yield Transcription Kit (Thermo Fisher). AECs lysates were freshly prepared using RIPA lysis buffer with proteinase inhibitor. The freshly prepared cell lysates (500 μg total protein) were incubated with the indicated biotinylated probes (500 ng) and 100 μM S‐adenosylmethionine (SAM, NEB, USA) in TENT buffer (20 mM Tris–HCl pH = 7.5, 250 mM NaCl, 1 mM EDTA, 0.5% TritonX‐100) supplemented with RNase inhibitor (0.5 U/μL, NEB) and protease inhibitor cocktail (Roche) at 37°C for 60 min with gentle rotation. Subsequently, the reaction mixture was isolated using Dynabeads M‐280 Streptavidin (Thermo Fisher), and the proteins were subjected to western blotting. For dot‐blot analysis, the beads‐RNA‐Protein complexes were subjected to RNA isolation using Trizol, and the RNA was denatured at 65°C for 10 min and spotted onto positively charged nylon membranes. M6A methylation level of *TERC* probes was assessed using m6A antibody and visualized by chemiluminescence. Subsequently, the membrane was stripped and subjected to streptavidin‐HRP antibody to detect the loading control.

For UV‐crosslinking RNA immunoprecipitation (RIP), cells were exposed to UVC (400 mJ/cm^2^) for crosslinking, followed by lysis using RIP Lysis buffer (50 mM HEPES pH 7.5, 150 mM KCl, 2 mM EDTA, and 1% NP‐40) supplemented with RNase inhibitor (0.5 U/μL, NEB) and protease inhibitor cocktail (Roche). Lysates were clarified by centrifugation (16,000 × *g*, 15 min at 4°C) and pre‐cleared with Protein A/G beads (Thermo fisher). Immunoprecipitations were performed overnight at 4°C with 3–5 μg of target‐specific antibodies or species‐matched IgG controls, followed by incubation with Protein A/G magnetic beads for 4 h. The IP materials were washed with IP wash buffer (100 mM Tris–HCl pH 7.4, 500 mM LiCl, 0.1% TritonX‐100, and 1 mM DTT) for three times, and RNA was extracted using Trizol (Vazyme, China) and assessed by RT‐qPCR. Enrichment was calculated as fold‐change relative to IgG controls after normalization to input RNA.

### 2.8 MeRIP‐qPCR

The meRIP‐qPCR assay was performed according to the standard protocol. Briefly, 10–20 μg RNA was fragmented into 200–300 nt fragments via a 15‐min incubation at 70°C in fragmentation buffer (10 mM ZnCl_2_, and 10 mM Tris–HCl pH 7.0). For exogenous *TERC* methylation analysis, RNA fragmentation was omitted to preserve full‐length transcripts. Thereafter, the fragmented RNA was incubated with 2–3 μg m6A antibody (Synaptic Systems) at 4°C overnight, and then the complex was incubated with DynaGreen Protein A/G magnetic beads (Thermo Fisher) at 4°C for 1 h. After washing with meRIP lysis buffer (150 mM NaCl, 0.1% NP‐40, and 10 mM Tris–HCl), the bound RNAs were recovered by proteinase K digestion, phenol–chloroform extraction, and ethanol preparation. One‐tenth of the fragmented RNA was used as an input control. The input or bound RNA was subjected to reverse transcription and subsequent qPCR. Relative enrichment was calculated as fold‐change relative to IgG controls after normalization to input RNA.

### 2.9 Subcellular Fractionation of Cytoplasmic and Nuclear Proteins

Cytoplasmic and nuclear proteins were extracted using a commercial subcellular protein fractionation kit (Beyotime Biotechnology). Briefly, cells were harvested, washed with ice‐cold PBS, and lysed in Cytoplasmic Lysis Buffer on ice for 15 min. The lysates were centrifuged at 12,000 × *g* for 5 min at 4°C. The supernatant (cytoplasmic fraction) was collected. The resulting pellet (nuclei) was further extracted with Nuclear Extraction Buffer containing on ice for 30 min. The nuclear extract was clarified by centrifugation at 12,000 × *g* for 10 min. Protein concentrations were determined using the BCA assay. The purity of the fractions was verified by Western blotting using antibodies against GAPDH (cytoplasmic marker) and Histone 3 (nuclear marker). RNA and proteins were extracted by Trizol (Invitrogen), according to the manufacturer's instructions. Quantification of the *TERC* in cytoplasmic and nuclear fractions was performed with specific primers.

### 2.10 Co‐Immunoprecipitation (CO‐IP)

Cells were lysed in IP lysis buffer (150 mM NaCl, 25 mM Tris–HCl pH 7.5, 1 mM EDTA, 0.5% NP‐40, 5% glycerol, and protein inhibitor). After centrifugation (16,000 × *g*, 30 min, 4°C), 500 μg lysate was immunoprecipitated overnight at 4°C with 3 μg antibody and Protein A/G beads. For RNase treatment, lysates were incubated with RNase A/T1 (100 μg/mL and 1000 U/mL respectively) at 37°C for 30 min prior to IP. The mixture was washed four times with the IP lysis buffer. The beads were resuspended in protein‐loading buffer and subjected to western blotting analysis.

### 2.11 Immunofluorescence Assay

Cells were fixed with 4% paraformaldehyde for 15 min and permeabilized in PBST (0.25% Triton X‐100) for 10 min at room temperature. After washing with PBS, the cells were blocked with 5% BSA for 30 min and incubated with the following antibodies: Flag (1:200; Sigma‐Aldrich, Germany), YTHDF1 (1:200; Proteintech, USA), YTHDF2 (1:200; Proteintech), YTHDF3 (1:200; Santa Cruz Biotechnology, USA), YTHDC1 (1:200; Cell Signaling Technology, USA), YTHDC2 (1:200; Proteintech), or coilin (1:200; Proteintech) overnight at 4°C. After washing three times with PBS, the cells were incubated with secondary antibodies (FITC/TRITC, 1:200, 1 h), followed by DAPI counterstaining. Imaging was performed on a Leica DMi8 (Germany) using standardized parameters.

### 2.12 Cell Proliferation, Cell Cycle Assay, EdU Incorporation, and SA‐β‐Gal

Cell proliferation and cell cycle assays were conducted as previously described (Cheng et al. 2021). EdU incorporation was performed using a kit (RiboBio, Guangzhou, China) following the manufacturer's instructions. SA‐β‐gal activity was assessed using a senescence β‐Galactosidase Staining Kit (GENMED Scientifics Inc., USA).

## 3 Statistical Analysis

Statistical analyses were performed using GraphPad software (version 8.0). All data were presented as mean ± standard deviations (SDs), unless otherwise stated. Normality of data was evaluated employing the Shapiro–Wilk test. For comparisons between two groups, two‐tailed unpaired Student's *t*‐tests were used if the normal distribution was satisfied; otherwise, the non‐parametric Mann–Whitney *U* test was applied. Meanwhile, the homogeneity of variance test was assessed using the *F*‐test. If the variances were homogeneous, an unpaired *t‐test* was employed; otherwise, the Welch's corrected *t‐test* was utilized. For multiple group comparisons involving more than two groups, one‐way or two‐way ANOVA followed by Tukey post hoc test was performed when normal distribution was satisfied; otherwise, the non‐parametric Kruskal‐Wallis test was conducted for further analysis. P values less than 0.05 were considered statistically significant.

## 4 Results

### 4.1 *TERC* RNA Undergoes Methylation at the N6‐Adenosine (m6A) Position of Both A111 and A435

**FIGURE 1:**
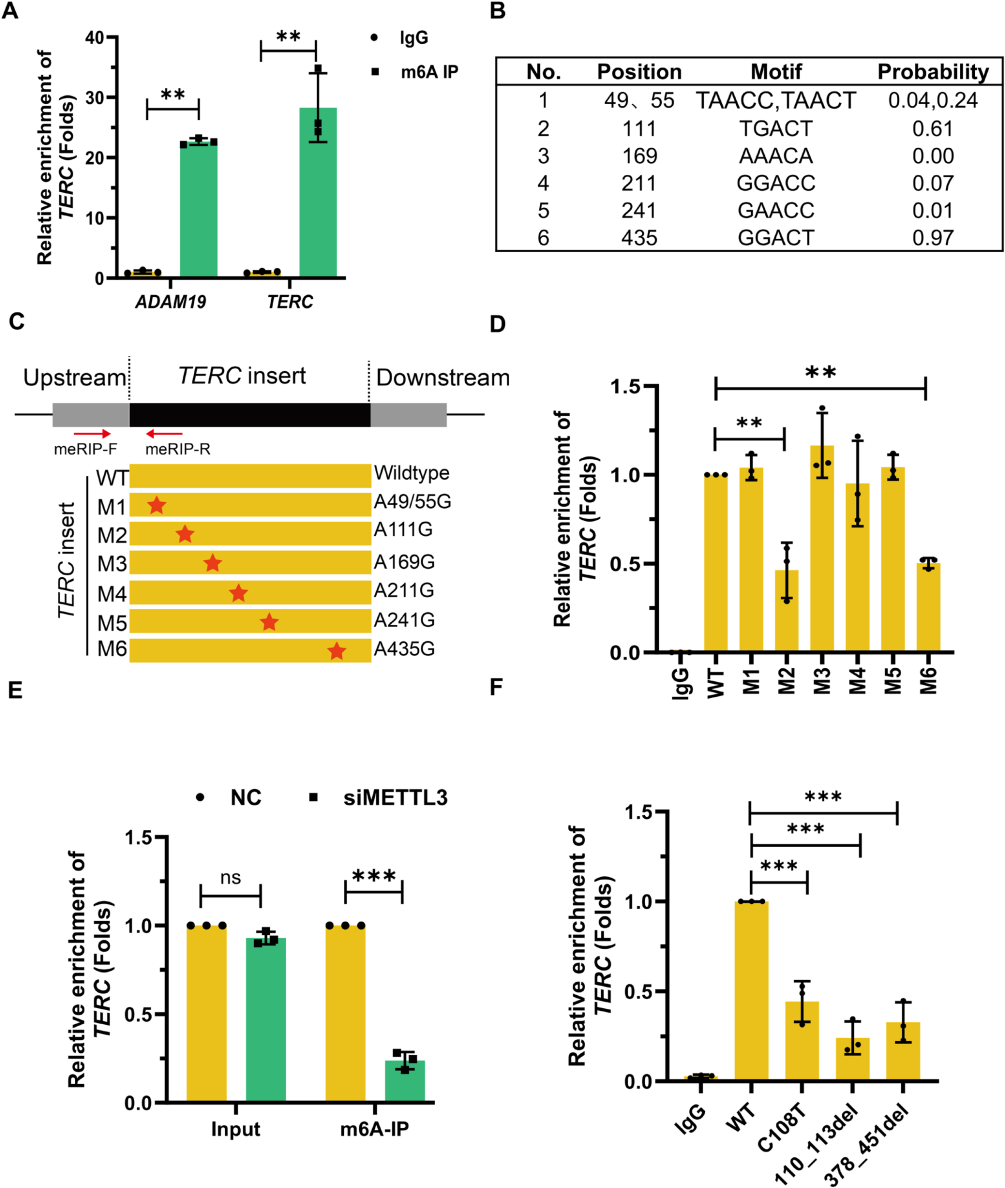
METTL3 mediates m6A methylation of *TERC* RNA. (A), MeRIP‐qPCR analysis of endogenous *TERC* RNA, showing significant m6A enrichment compared to the negative control (IgG). ADAM, positive control. **, *p* < 0.01. (*n* = 3). (B), Putative m6A methylation sites in TERC RNA predicted by deepSRAMP algorithm. (C), Schematic graphic depicted the putative m6A methylation site and designed mutant transcript. Red arrows indicated primer pairs (listed in Table S1) for exogenous *TERC* detection in m6A‐ RIP‐qPCR. (D), *TERC* wild‐type and mutant transcripts mentioned in D were transfected into AECs. Forty‐eight hours later, total RNA was extracted and subjected to MeRIP‐qPCR to examine the m6A methylation level of exogenous *TERC*. Fold enrichment was normalized to input and WT controls. **, *p* < 0.01. (*n* = 3). (E), METTL3 knockdown significantly reduced m6A modification on *TERC* RNA, as assessed by MeRIP‐qPCR. ***, *p* < 0.001. (*n* = 3). (F), MeRIP‐qPCR analysis reveals the distinct m6A methylation levels in PF‐associated *TERC* deletion variants. ***, *p* < 0.001. (*n* = 3).

To establish the presence of m6A modifications on *TERC*, we preliminarily performed the m6A‐RNA immunoprecipitation (MeRIP) assays. *TERC* showed significant enrichment with m6A antibody compared to IgG control (Figure 1A), similar to the positive control *ADAM19* mRNA. Although previous transcriptome‐wide studies suggested A435 as the primary m6A site, public nanopore sequencing data (DirectRMDB) indicated A111 and A241 as potential sites. To resolve this discrepancy, we designed experiments to screen and identify the actual methylation sites. Since N6‐Methyladenosine preferentially occurs at the consensus motif DRACH (R is A or G; H is U, A, or C) (Nombela et al. 2021), we used the deepSRAMP tool (A sequence‐based m6A site predictor by a combined deep learning framework) to screen and speculate on the potential m6A methylation site (Figure 1B). Based on the computational screening, we generated wild‐type (WT) and mutant *TERC* constructs (Figure 1C) by subcloning the corresponding sequences into the pcDNA3.1(+) plasmid. These plasmids were transfected into AECs, and after 48 h, total RNA was extracted and subjected to MeRIP‐qPCR to assess the m6A methylation levels in exogenous *TERC*. MeRIP analysis revealed a significant reduction in m6A enrichment upon mutation of either A111 or A435 (Figure 1D), confirming these as major methylation sites. Strikingly, the double mutant (A111G/A435G, M2/M6) exhibited an even more pronounced decrease in m6A methylation (Figure S1A,B). Meanwhile, METTL3 deficiency dramatically decreased the enrichment of *TERC* (Figure 1E), confirming METTL3‐dependent methylation. Notably, mutations near the methylation sites (C108T, r.110_113delGACU, r.378_451delGACU) are closely related to pulmonary fibrosis, although their mechanistic contribution remains unclear. We engineered *TERC* transcripts harboring the disease‐associated variants (Figure S1A). Strikingly, these mutants exhibited a significant reduction in m6A methylation levels (Figure 1F), implicating m6A dysregulation as a potential contributor to disease pathogenesis. Interestingly, the methylation sites are highly conserved across mammals (Figure S1C), suggesting that m6A methylation at these positions may play a critical and evolutionarily conserved role in *TERC* function. Collectively, these findings clearly state that METTL3‐mediated m6A modification occurs in *TERC* RNA with A111 and A435 serving as the primary methylation sites.

### 4.2 Loss of m6A Modification in *TERC* Leads to Impaired Telomerase Activity and Progressive Telomere Shortening

**FIGURE 2:**
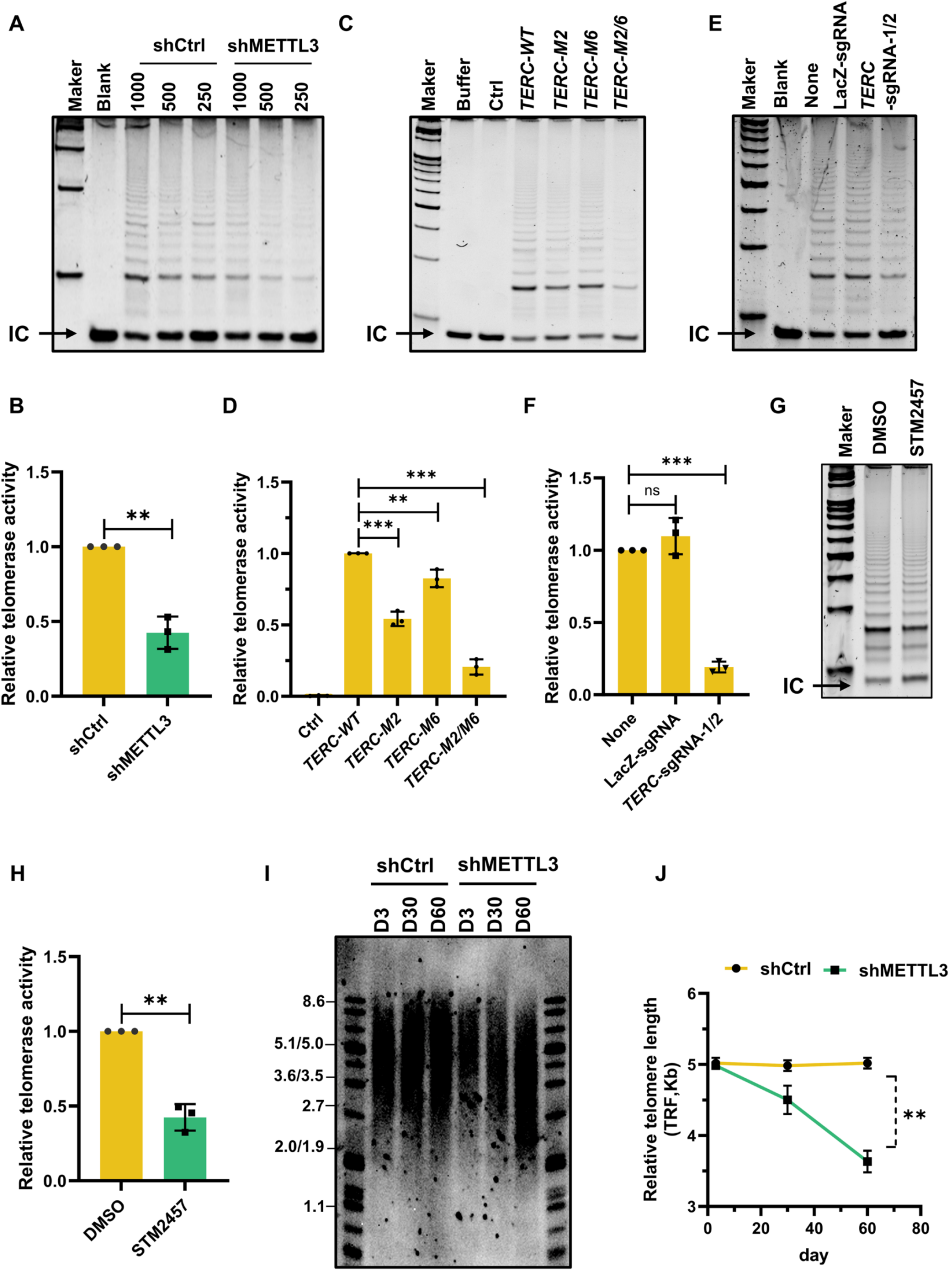
m6A‐modified *TERC* promotes telomerase activity and telomere maintenance. (A, B), AECs stably expressing Control (shCtrl) or METTL3 shRNA (shMETTL3) were subjected to TRAP assays to evaluate the telomerase activity. **, *p* < 0.01. (*n* = 3). (C, D), U2OS cells were co‐transfected with Flag‐TERT and wild‐type *TERC* or m6A site mutant Transcript. Forty‐eight hours later, TRAP assays were performed to determine the telomerase activity. **, *p* < 0.01; ***, *p* < 0.001. (*n* = 3). (E, F) TRAP assay assessment of telomerase activity following *TERC*‐targeted m6A demethylation using the dCasRx‐ALKBH5 system. ***, *p* < 0.001. (*n* = 3). (G, H), AECs were treated with DMSO or STM2457, a specific inhibitor of METTL3. Forty‐eight hours later, TRAP assays were performed to determine the telomerase activity. **, *p* < 0.01. (*n* = 3). (I), AECs stably expressing Control (shCtrl) or METTL3 shRNA (shMETTL3) were passaged with time and the average telomere length was assessed using Southern blot. (J), Quantification of telomere length from G, demonstrating significant shortening upon METTL3 depletion. **, *p* < 0.01. (*n* = 3).

While a prior study reported that ALKBH5 overexpression reduces telomerase activity by 22% (Han et al. 2020), the mechanistic basis for this observation remained unclear. To rigorously investigate the role of m6A methylation in telomerase function, we generated AECs with stable METTL3 knockdown (Figure S1D). Strikingly, telomerase activity decreased by 57.5% in METTL3‐depleted cells compared to controls, as measured by TRAP assay (Figure 2A,B). To further dissect the role of *TERC* m6A methylation, we utilized U2OS cells—a well‐established reconstitution system lacking endogenous TERT and *TERC* (Tang et al. 2018). When co‐transfected with Flag‐TERT and either wild‐type or methylation‐deficient mutant *TERC* (M2 [A111], M6 [A435], or double mutant), we observed a moderate reduction in telomerase activity with single‐site mutants (A111 or A435) and a dramatic impairment in the double mutant condition (Figure 2C,D). This dose‐dependent effect aligns with the progressive reduction in m6A methylation observed in these mutants (Figure 1D, Figure S1B). Expression levels of *TERC* and Flag‐TERT were confirmed by real‐time qPCR and Western blotting (Figure S2A,B). To rule out the potential impact of mutagenesis on *TERC* structure, we employed a dCasRx‐alkbh5 RNA editing system to selectively erase m6A methylation on *TERC*. The results suggested that the *TERC*‐targeting sgRNA‐guided dCasRx‐ALKBH5 system efficiently demethylated the *TERC*, but not ADAM19 (Figure S2C,D). Strikingly, *TERC* demethylation recapitulated the telomerase activity defect observed in methylation‐site mutants (Figure 2E,F), confirming that the phenotype stems from m6A loss rather than structural perturbations. To ascertain the requirement for METTL3's catalytic activity, we used its specific inhibitor, STM2457, which is currently in clinical trials. STM2457 treatment potently suppressed global RNA m6A methylation, as measured by RNA dot blot, while METTL3 protein levels were largely unaffected (Figure S2E). Consequently, telomerase activity was severely impaired (Figure 2G,H). To assess long‐term functional consequences, we performed Southern blotting in METTL3‐depleted cells, which exhibited a 30% reduction in mean telomere length (Figure 2I,J). Meanwhile, we performed longitudinal telomere length analysis in AECs transfected with control (pcDNA), wild‐type *TERC*, or mutant *TERC* plasmids every 3 days, with samples collected on the indicated days for Southern blot analysis. Remarkably, cells expressing wild‐type *TERC* exhibited progressive telomere elongation, while those with mutant *TERC* showed minimal length changes (Figure S2F,G). Collectively, these results provide compelling evidence that m6A modification of *TERC* is critical for maintaining telomerase activity and telomere length homeostasis.

### 4.3 *TERC* m6A Modification Facilitates TERT‐ *TERC* Association

**FIGURE 3:**
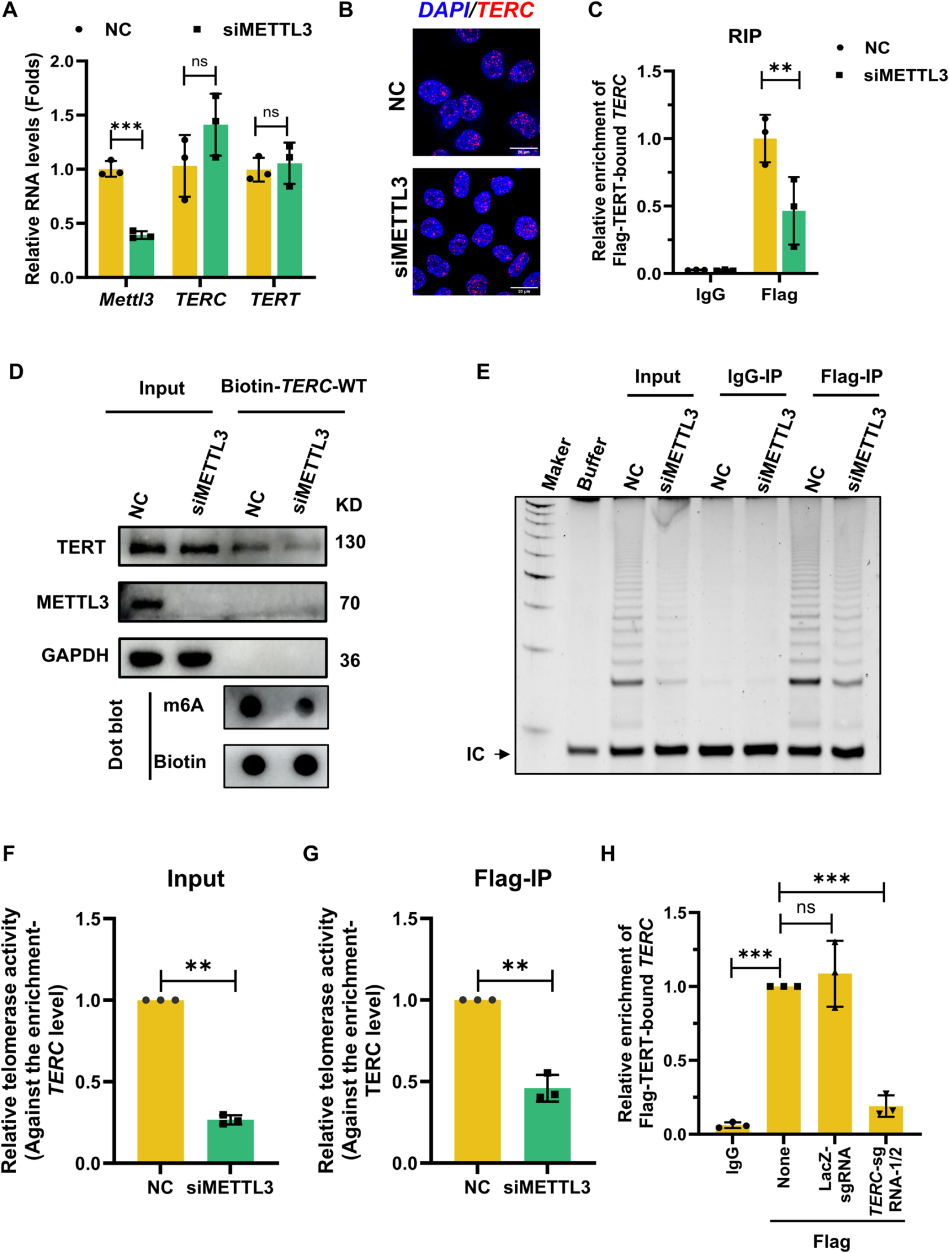
m6A‐modified *TERC* strengthens TERT‐*TERC* assembly. (A), RT‐qPCR analysis of *TERC* and *TERT* mRNA levels in METTL3‐knockdown AECs. ***, *p* < 0.001. (*n* = 3). (B), RNAscope assay was performed to assess the subcellular localization of *TERC* upon METTL3 knockdown. Red, *TERC*; Blue, DAPI. Scale bar, 20 μm. (C), AECs stably expressing Flag‐TERT were transfected with control siRNA (NC) or METTL3 siRNA (siMETTL3). Subsequently, cells were exposed to UV crosslinking followed by RNA immunoprecipitation (RIP) with a Flag antibody to analyze the combination of Flag‐TERT with *TERC*. **, *p* < 0.01. (*n* = 3). (D), RNA pulldown assay using biotin‐labeled *TERC* probes in AECs transfected with control (NC) or METTL3 siRNA (siMETTL3) was performed to evaluate the association of TERT and *TERC*. Top: Western blotting of TERT in the pulldown material; Bottom: RNA dot blot confirming m6A levels in probes. (E), AECs stably expressing Flag‐TERT were transfected with control siRNA (NC) or METTL3 siRNA (siMETTL3). Forty‐eight hours later, cells were subjected to IP assay using a Flag antibody. The IP materials were subjected to assess the telomerase activity using TRAP assay. (F, G), Data from E were quantified against to the *TERC* expression level in IP products. **, *p* < 0.01. (*n* = 3). (H), AECs stably expressing Flag‐TERT were treated with *TERC*‐targeted m6A demethylation using the dCasRx‐ALKBH5 system. Subsequently, the cells were exposed to UV crosslinking followed by RIP with a Flag antibody to analyze the combination of Flag‐TERT with *TERC*. ***, *p* < 0.001. (*n* = 3).

To elucidate the mechanism by which *TERC* m6A modifications regulate telomerase activity, we first examined *TERC* and TERT expression levels, finding only minor alterations upon METTL3 knockdown (Figure 3A). Furthermore, to determine whether METTL3 knockdown affects *TERC* localization, we used RNAscope and cellular fractionation assays. RNAscope analysis revealed that *TERC* was primarily localized in the nucleus, and this distribution pattern remained largely unaltered upon METTL3 silencing (Figure 3B). This observation was corroborated by detecting TERC in separated cytoplasmic and nuclear fractions (Figure S3A,B). However, immunofluorescence revealed slightly reduced co‐localization of TERT with Cajal bodies (marked by Coilin) following METTL3 depletion (Figure S3C), hinting that defective holoenzyme assembly. To further confirm our hypothesis, we conducted immunoprecipitation assays and modified RNA pulldown. The *TERC* level in Flag‐TERT‐enriched materials decreased upon METTL3 knockdown (Figure 3C). Meanwhile, RNA pulldown assays further demonstrated weakened TERT binding to m6A‐deficient *TERC* upon METTL3 knockdown, corroborated by reduced m6A signals in biotin‐labeled *TERC* via RNA dot blot (Figure 3D). Consistent with these findings, RNA pulldown with biotin‐labeled wild‐type or mutant *TERC* (M2/M6), the association of TERT with mutant *TERC* was dramatically decreased compared with the wild‐type *TERC* (Figure S3B). Additionally, IP‐TRAP assays were performed as previously described (Tang et al. 2018; Ford et al. 2000). METTL3 knockdown resulted in decreased enrichment of the telomerase holoenzyme immunoprecipitated with Flag‐TERT (Figure 3E), indicating that m6A methylation facilitates the TERT‐*TERC* interaction. To compare the catalytic activity of m6A‐modified versus unmethylated telomerase, we quantified its specific activity by normalizing the TRAP signal from the immunoprecipitated complexes to the levels of co‐precipitated *TERC*. The normalized activity of telomerase was also decreased upon METTL3 depletion, implying that the holoenzyme containing m6A‐modified TERC possesses higher intrinsic catalytic activity (Figure 3F,G, Figure S3E,F). Furthermore, the *TERC*‐targeting sgRNA‐guided dCasRx‐ALKBH5 system significantly impaired the interaction between Flag‐TERT and *TERC* (Figure 3H). In summary, our findings imply that m6A modification of *TERC* maintains telomerase activity by facilitating the assembly of TERT and m6A‐methylated *TERC*.

### 4.4 YTHDC1 Binds to *TERC* via Recognizing m6A Sites of *TERC* RNA

**FIGURE 4:**
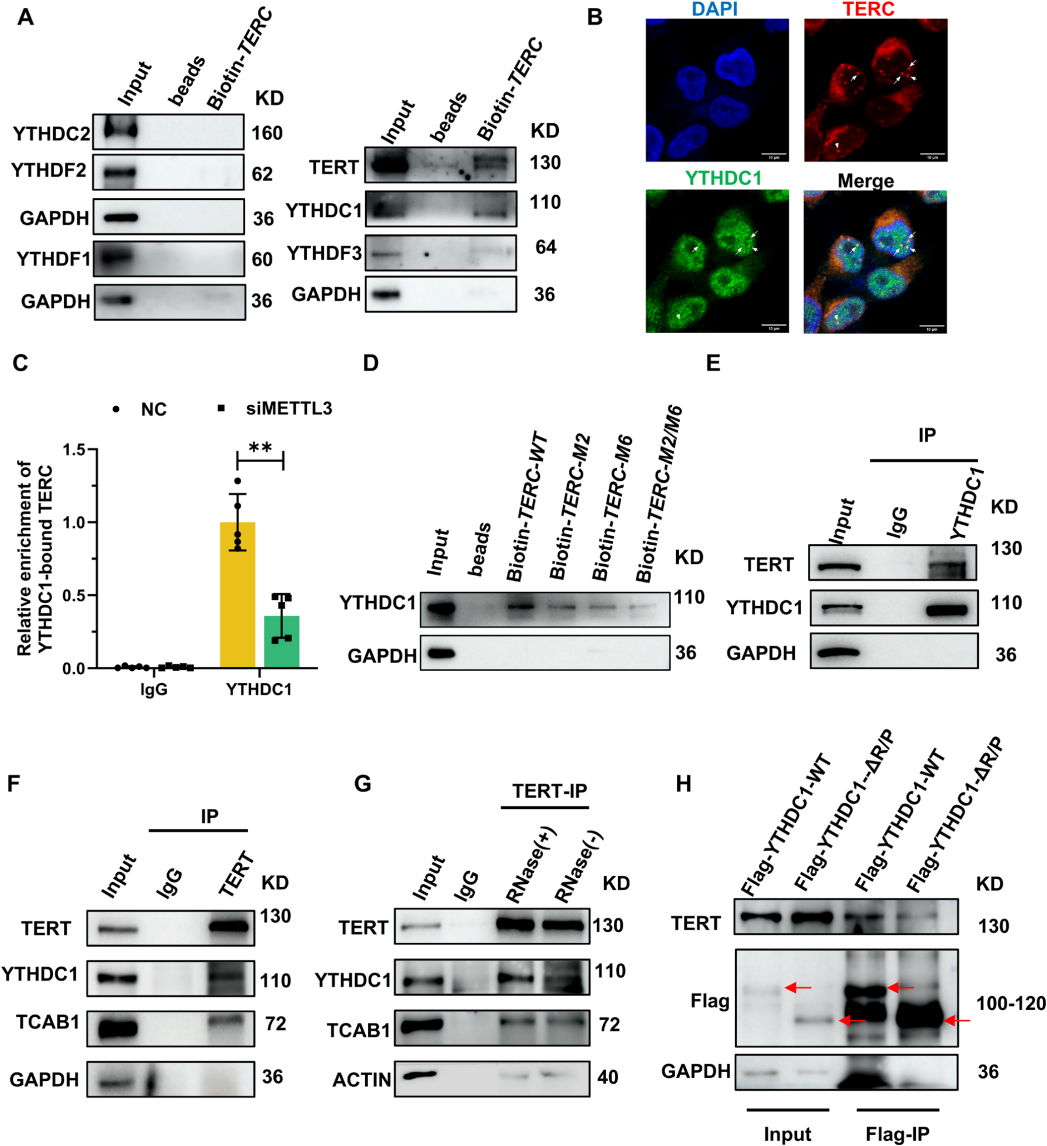
YTHDC1 binds to m6A‐modified *TERC* or TERT. (A), AEC cell lysates were subjected to RNA pulldown with biotin–labeled *TERC* probe, and the pulldown materials were analyzed by western blotting. (B), RNAscope combined with immunofluorescence assay demonstrating the co‐localization of *TERC* RNA (red) and YTHDC1 (green). Scale bar: 10 μm. (C), AECs were transfected with control (NC) or METTL3 siRNA (siMETTL3), forty‐eight hours later, cells were subjected to UV‐crosslink followed by RIP using YTHDC1 antibody to assess the association of YTHDC1 and *TERC*. **, *p* < 0.01. (*n* = 5). (D), RNA pulldown using biotin‐labeled wild‐type or mutant *TERC* probe (M2, M6 or both) was performed to assess the YTHDC1‐*TERC* interaction. (E, F), AECs stably were subjected to immunoprecipitated using a YTHDC1 (E) or TERT (F) antibody, and the IP materials were assessed using western blotting for indicated proteins. lgG was used as a negative control. (G), IP was performed in above cells using TERT antibody in the presence or absence of RNase A/T1 treatment. (H), AECs transfected with Flag‐YTHDC1‐WT or Flag‐YTHDC1‐ΔR/P were subjected to immunoprecipitated using a Flag antibody, the IP materials were assessed using western blotting for indicated proteins.

Previous studies have established that the assembly and activity of the telomerase holoenzyme depend on dyskerin, GAR1, NOP10, and NHP2, which bind directly to *TERC* (Schmidt and Cech 2015). However, we observed that neither the expression levels of these proteins nor their association with TERC was affected by METTL3 knockdown (Figure S3G,H). We therefore turned our attention to YTH domain‐containing reader proteins, which are known to mediate m6A‐dependent RNA–protein interactions (Boulias and Greer 2023; Zhao et al. 2017), as potential alternative regulators. Bioinformatics analysis using POSTAR3 (Zhao et al. 2022) predicted YTHDC1 as a putative *TERC*‐binding protein (Table S2). RNA pulldown and immunoprecipitation (RIP) assays confirmed YTHDC1 as the predominant m6A reader protein interacting with *TERC* (Figure 4A and Figure S4A). Furthermore, IP‐TRAP assays showed that, similar to Flag‐TERT, YTHDC1 co‐precipitated with the telomerase holoenzyme (Figure S4B,C), corroborating that YTHDC1 may bind to telomerase RNA *TERC*. Additionally, immunofluorescence and RNAscope demonstrated YTHDC1 co‐localization with both coilin (a Cajal body marker) and *TERC* itself (Figure 4B and Figure S4D). Unsurprisingly, RIP assays revealed that METTL3 knockdown reduced YTHDC1 binding to *TERC* (Figure 4C). RNA pulldown experiments using biotin‐labeled *TERC* transcripts (wild‐type, M2 [A111], M6 [A435], or double mutant) further showed impaired YTHDC1 binding with *TERC* upon mutation of the methylation sites (Figure 4D). Notably, single‐site mutants exhibited partial reductions in binding, while the double mutant showed near‐complete loss of YTHDC1 interaction. Therefore, these data indicate that YTHDC1 participates in telomerase holoenzyme assembly by binding to *TERC*, a process that is influenced by the m6A methylation status of *TERC*.

### 4.5 YTHDC1 Directly Associates With TERT in an RNA‐Independent Manner

Based on the finding that *TERC* m6A modification facilitates TERT‐*TERC* association, we hypothesized that YTHDC1 mediates this interaction. To test this, we performed co‐immunoprecipitation (co‐IP) assays using antibodies against YTHDC1 or TERT. As shown in Figure 4E,F, western blotting revealed that TERT associated with YTHDC1. Importantly, this interaction persisted after RNase A/T1 treatment (Figure 4G), demonstrating it occurs in an RNA‐independent manner. Consistent with this finding, YTHDC1 also co‐immunoprecipitated with Flag‐tagged TERT in *TERC*‐deficient U2OS cells (Figure S5A), further demonstrating that the YTHDC1‐TERT interaction occurs independently of *TERC*. As reported previously, YTHDC1 comprises six regions and one classic YTH domain responsible for binding m6A‐modified RNA (Dattilo et al. 2023). To identify the TERT‐binding domain of YTHDC1, we generated a series of Flag‐tagged truncation mutants encompassing the six structural regions of YTHDC1 (Figure S5B). Co‐IP experiments showed that wild‐type YTHDC1, but not deletion mutants Δ4, Δ5, or Δ6, maintained the ability to bind TERT (Figure S5C). This identified amino acids 345‐648 (encompassing the YTH and R/P domains) as the critical interaction domain. Intriguingly, a mutant YTHDC1 lacking the R/P domain also exhibited weakened TERT binding while maintaining its association with *TERC* (Figure 4H and Figure S5D). Additionally, deletion of the YTH domain severely compromised the interaction with TERC (Figure S5E), confirming that the YTH domain is essential for recognizing m6A‐modified *TERC*. Furthermore, the TERT‐YTHDC1 interaction was unaffected by METTL14 knockdown (Figure S5F). Collectively, these data demonstrate that YTHDC1 directly binds TERT through its C‐terminal domain (amino acids 345‐648) in a *TERC*‐independent manner.

### 4.6 YTHDC1 Scaffolds *TERC* ‐TERT Interaction in Telomerase Assembly

**FIGURE 5:**
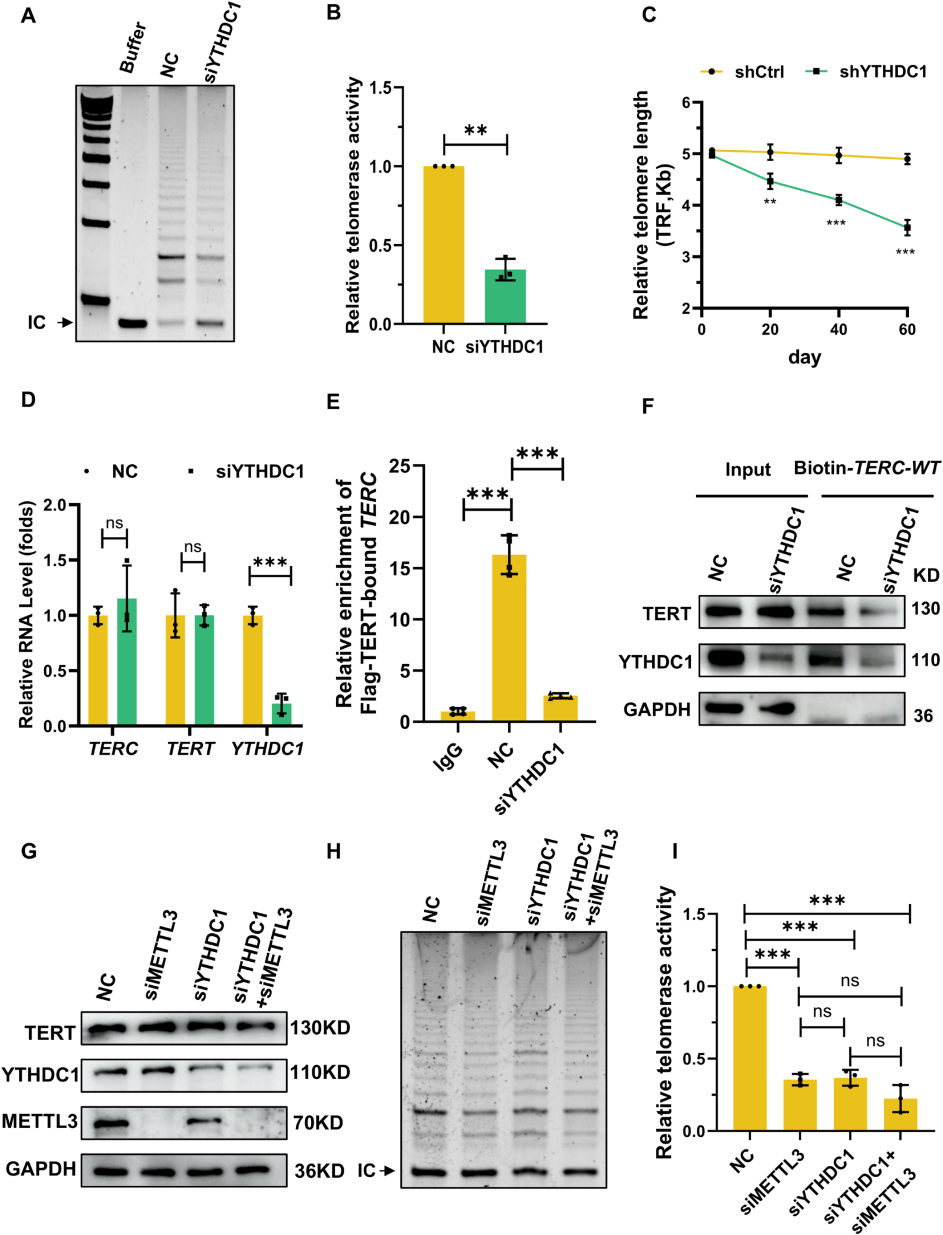
YTHDC1 scaffolds *TERC*‐TERT interaction in telomerase assembly. (A‐B), AECs were transfected with a control (NC) or YTHDC1‐targeting siRNA (siYTHDC1). Forty‐eight hours later, TRAP assays were conducted to assess the telomerase activity. **, *p* < 0.01. (*n* = 3). (C), Telomere length analysis by Southern blot in control (shCtrl) versus YTHDC1‐knockdown (shYTHDC1) AECs over serial passages. **, *p* < 0.01; ***, *p* < 0.001. (*n* = 3). (D), RT‐qPCR analysis of *TERC* and *TERT* mRNA levels in YTHDC1‐knockdown AECs. ***, *p* < 0.001. (*n* = 3). (E), AECs stably expressing Flag‐TERT were transfected with NC siRNA or YTHDC1 siRNA. The cells described above were subjected to UV crosslinking followed by RIP with a Flag antibody to analyze the combination of Flag‐TERT with *TERC*. ***, *p* < 0.01. (*n* = 3). (F), AECs were transfected with a siRNA targeting YTHDC1. Forty‐eight hours later, RNA pulldown was performed with cell lysates and biotin‐labeled *TERC* probes, the presence of TERT in pulldown materials was assessed by western blotting. (G), AECs were transfected with control (NC), METTL3 (siMETTL3), YTHDC1 (siYTHDC1), or a combination of METTL3 and YTHDC1 siRNAs (siYTHDC1+ siMETTL3). The cells were subjected to western blotting. (H, I), The cells described in G were collected and subjected to TRAP assays. ***, *p* < 0.001. (*n* = 3).

To dissect YTHDC1's functional role, we first assessed its effect on telomerase activity. TRAP assays revealed a striking 65.6% reduction in telomerase activity upon YTHDC1 knockdown (Figure 5A,B), accompanied by progressive telomere shortening in YTHDC1‐deficient cells (Figure 5C and Figure S6A). Crucially, similar to METTL3 depletion, YTHDC1 knockdown did not alter *TERC*/TERT transcript or TERT protein levels, hinting that YTHDC1 might also affect telomerase assembly (Figure 5D,G). Supporting this, IP‐TRAP assays showed significantly diminished telomerase enrichment by Flag‐TERT in YTHDC1‐deficient cells (Figure S6B,C). Furthermore, RNA immunoprecipitation and RNA pulldown experiments demonstrated that YTHDC1 knockdown weakened the TERT‐*TERC* association (Figure 5E,F), phenocopying the effect of METTL3 depletion. Notably, although YTHDC1 knockdown did not alter the expression of the core telomerase‐associated proteins dyskerin, GAR1, NOP10, or NHP2, it significantly impaired the association between TERT and these *TERC*‐binding factors (Figure S6D). These results further support the conclusion that YTHDC1 influences telomerase holoenzyme assembly. To determine whether YTHDC1's effect on telomerase activity depends on m6A methylation, we silenced the expression of YTHDC1, METTL3,or both. Western blotting confirmed that TERT protein levels remained unchanged under all conditions (Figure 5G). Strikingly, while YTHDC1 depletion alone reduced telomerase activity, its knockdown in METTL3‐deficient cells failed to cause a further suppression (lane 2 vs. lane 4, Figure 5H,I). This indicates that YTHDC1's regulation of telomerase is strictly m6A‐dependent. Taken together, these data support the hypothesis that YTHDC1 promotes telomerase holoenzyme assembly by strengthening the combination of m6A‐methylation *TERC* with TERT.

### 4.7 YTHDC1‐Mediated Telomerase Assembly Enhances Proliferation and Antagonizes Senescence in AECs

**FIGURE 6:**
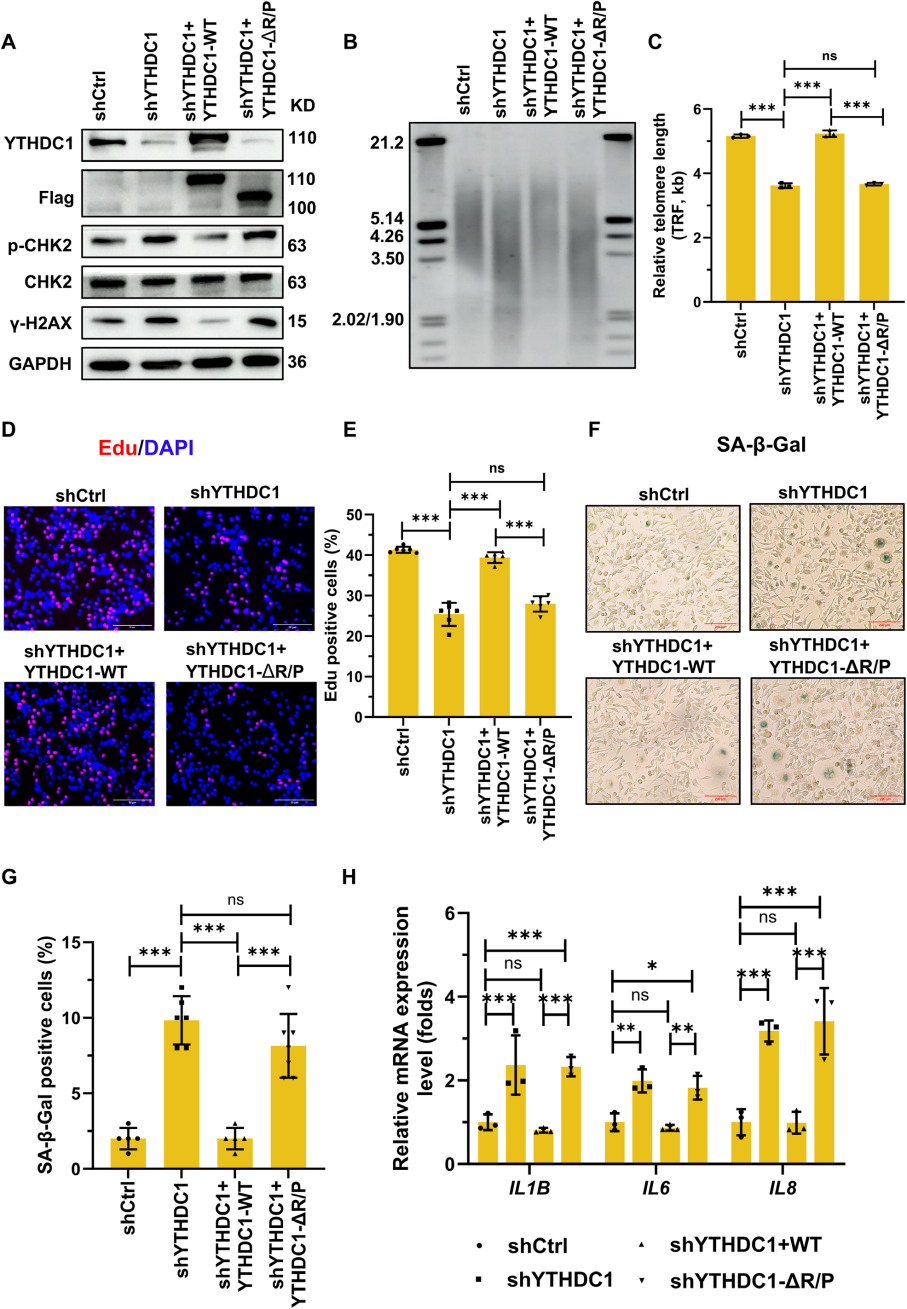
YTHDC1‐mediated telomerase assembly enhances proliferation and antagonizes senescence in AECs. (A) AECs with stable YTHDC1 knockdown were transfected with YTHDC1‐WT or YTHDC1‐ΔR/P plasmids every 3 days, and the cells were subjected to western blotting. (B) The cells described in A were cultured for the indicated times and subjected to southern blot to assess the average telomere length. (C) Telomere length data from B were quantified. **, *p* < 0.01. (*n* = 3). (D, E) Cells from A were subjected to EdU incorporation assays to evaluate the cell proliferation. ***, *p* < 0.001. (*n* = 6). (F, G) Cells described in A were exposed to SA‐β‐gal staining. Representative images and the quantitative analysis are shown. ***, *p* < 0.001. (*n* = 6). (H) RNA isolated from AECs described in A was subjected to RT‐qPCR to assess the *IL1B*, *IL6*, and *IL8* mRNA expression levels. *, *p* < 0.05; **, *p* < 0.01; ***, *p* < 0.001. (*n* = 3).

Given the established role of telomerase activity in maintaining cellular proliferation and preventing senescence (Blackburn et al. 2015; Shawi and Autexier 2008; Schmidt and Cech 2015), we investigated whether YTHDC1‐mediated telomerase assembly impacts these processes in AECs. To this end, we performed rescue experiments in YTHDC1‐deficient AECs by continuously transfecting plasmids expressing either wild‐type YTHDC1 or a truncated mutant (Flag‐YTHDC1‐ΔR/P, lacking the critical TERT‐binding domain) every 3 days until population doubling 60 (PD60). Since telomere dysfunction or telomerase deficiency is known to activate DNA damage signaling, we first assessed the levels of γ‐H2AX and p‐CHK2, both markers of DNA damage. YTHDC1 knockdown increased the expression of these markers. However, this effect was reversed by expression of wild‐type YTHDC1, but not the truncated mutant YTHDC1 (Figure 6A and Figure S7A). Consistently, expression of wild‐type YTHDC1, but not the mutant, rescued the loss of telomerase activity and telomere length erosion (Figure S7B,C and Figure 6B,C). We next examined cellular proliferation and senescence using EdU incorporation assays (Figure 6D,E), ki67 immunofluorescence (Figure S7D,E), senescence‐associated β‐galactosidase assays (Figure 6F,G), senescence‐associated secretory phenotype (SASP) evaluation (Figure 6H), and cell cycle analysis (Figure S7F). As expected, YTHDC1 deficiency induced S phase arrest, decreased EdU incorporation, lower ki67‐positive rates, and accelerated cellular senescence (increased SA‐β‐gal positive staining and elevated SASP level). Crucially, these defects were rescued by wild‐type YTHDC1 expression, whereas the R/P domain‐truncated mutant failed to restore the proliferation or delay senescence. Altogether, these findings demonstrate that YTHDC1 promotes telomerase assembly by strengthening the TERT–*TERC* interaction, which in turn is critical for sustaining proliferation and suppressing senescence in AECs.

## 5 Discussion

Telomerase RNA *TERC*, as a core component of telomerase, provides the template for telomeric DNA synthesis through reverse transcription catalyzed by TERT. *TERC*'s secondary structure is extremely complex and contains pseudo/template areas that are critical to TERT binding (Pseudoknot/Template domain) and CR4/CR5 regions, as well as ScaRNA domains responsible for its processing and stability. While the association of multiple RNA binding proteins to *TERC* is indispensable for its maturation, structural maintenance, assembly, and subcellular localization, the RNA's own post‐transcriptional modifications also serve as key regulators of telomerase activity. Our previous work demonstrated that m5C methylation of *TERC* modulates telomerase activity by promoting the assembly of TERT and *TERC* (Tang et al. 2018). A recent study suggested that m6A modification occurs in *TERC* and regulates telomerase activity (Han et al. 2020), however, the mechanistic basis remained unresolved. In this study, we further establish that m6A methylation of *TERC* promotes telomerase activity and telomere maintenance, with disease‐associated *TERC* mutations significantly reducing methylation levels (Figures 1 and 2). In addition, we elucidate the underlying mechanism by which YTHDC1 functions as a critical adaptor that simultaneously binds TERT and m6A‐modified *TERC*, thereby bridging their interaction (Figures 3 and 4). Crucially, this direct YTHDC1‐TERT association is indispensable for enhancing telomerase activity, ultimately impacting cellular proliferation and senescence (Figures 5 and 6). These findings provide new insights into the role of RNA methylation in telomerase regulation and establish a novel mechanistic paradigm for YTHDC1‐dependent control of telomerase activity.

The functional outcomes of m6A‐modified RNAs are typically determined by reader proteins, such as YTH family proteins, which regulate RNA transcription, splicing, stability, and translation (Zaccara and Jaffrey 2020). However, *TERC* expression remained unchanged upon METTL3 knockdown (Figure 3A) or ALKBH5 overexpression (Han et al. 2020), indicating m6A does not regulate *TERC* stability. Oxidative stress, DNA damage, or viral infection are known to alter TERC trafficking, and METTL3 participates in these processes (Li et al. 2021; Zhang et al. 2020; Houmani and Ruf 2009; Cheng et al. 2018). However, METTL3 knockdown did not significantly affect TERC subcellular localization (Figure 3B and Figure S3A,B). This finding indicates that METTL3 is not an essential regulator of *TERC* trafficking during these events, which is likely governed primarily by other factors.

Previous evidence shows that TCAB1 knockdown disrupts *TERC* localization to Cajal bodies (Venteicher et al. 2009; Chen et al. 2018; Nguyen et al. 2018). Notably, our data reveal that *TERC* m6A methylation levels are unaffected by TCAB1 depletion (Figure S8A,B), demonstrating that *TERC* methylation occurs prior to Cajal body entry. We therefore propose a novel assembly model wherein YTHDC1 facilitates the association of m6A‐modified *TERC* with TERT outside Cajal bodies, subsequently recruited to Cajal bodies via TCAB1. Consistent with this model, METTL3 knockdown resulted in TERT accumulation outside Cajal bodies (Figure S3C). This regulatory mechanism warrants further investigation.

A prior study suggested ALKBH5 overexpression disrupts TCAB1‐DKC1 interaction, which suggested that ALKBH5 promotes the disassembly of telomerase (Han et al. 2020). However, this observation is controversial as TCAB1 loss reduces the rate of enzymatic activity without compromising RNP processability or the assembly of the catalytic core (Chen et al. 2018). Our work provides a new perspective suggesting that YTHDC1 functions as a scaffold for telomerase assembly. Beyond its canonical role as an RNA‐binding protein, YTHDC1 exhibits protein‐interaction capabilities (Dattilo et al. 2023; Yuan et al. 2023). Here, we demonstrate that YTHDC1's C‐terminal domain (aa 345–648) mediates direct, RNA‐independent binding to TERT—unaffected by METTL14 knockdown (Figure S5F). Although TERT has been reported to translocate to mitochondria under stress conditions (Ahmed et al. 2008). Knocking down YTHDC1 does not alter the subcellular localization of TERT (Figure S9). This indicated that while the two proteins’ interaction is important for telomerase holoenzyme assembly, it does not influence the mitochondrial translocation of TERT. The regulation of TERT's mitochondrial localization is therefore likely governed by factors independent of the YTHDC1 pathway. Together, these findings redefine YTHDC1 as a bifunctional adaptor critical for telomerase holoenzyme formation.

Previous studies implicate that METTL3‐mediated m6A modification in *TERRA* stabilization via YTHDC1, promoting ALT‐dependent telomere maintenance (Chen et al. 2022; Vaid et al. 2024). *TERRA* is an RNA copy of the C‐rich telomere DNA strand transcribed by RNA polymerase II. It binds to telomere DNA and is integral to the telomere structure, protecting telomere ends from damage and maintaining telomere length, integrity, and stability (Luke 2009; Yadav et al. 2022). Therefore, the regulation of telomerase activity and telomere length by METTL3 cannot be ruled out by *TERRA*. However, we employed dCasRx‐ALKBH5 techniques or introduced mutations at methylation sites to specifically remove m6A modifications on *TERC*. These approaches demonstrate that loss of *TERC* m6A modifications suffices to impair telomerase activity, highlighting the importance of *TERC* m6A modification. Crucially, a YTHDC1 truncation mutant (lacking the R/P domain but retaining the YTH domain) maintained binding to m6A‐modified *TERC* (and presumably other m6A‐RNAs like *TERRA*), yet failed to rescue telomerase activity in YTHDC1‐deficient cells. This definitively establishes that telomerase assembly requires YTHDC1's scaffolding function—bridging m6A‐*TERC* and TERT via its dual binding domains.

In summary, our study elucidates a novel mechanistic framework in which m6A methylation of *TERC* serves as a critical regulator of telomerase activity. We identify YTHDC1 as a crucial m6A reader that functions as a molecular scaffold to facilitate telomerase holoenzyme assembly, thereby promoting the proliferation of AECs (Graphical Abstract). These findings establish a direct link between RNA epitranscriptomic modifications and telomerase regulation, providing new insights into post‐transcriptional control of cellular proliferation.

## Author Contributions

X.G., H.T., J.X., W.W., and Z.M. conceived and designed the experiments. X.C., T.X., S.W., and Y.Y. performed the experiments. J.X., Q.W., Y.W., Z.J., and X.Q. collected the data. D.J., Y.S., X.Q., and Z.L. performed formal analysis and data curation. X.G., H.T., and X.C. wrote the paper. All authors read and approved the final manuscript.

## Funding

This work was supported by National Natural Science Foundation of China (82101636, 82371569), China Postdoctoral Science Foundation (2022M721572), Jiangsu Funding Program for Excellent Postdoctoral Talent (2022ZB703). Henan Provincial Joint Fund of Science and Technology Research and Development Program (225200810115), Henan Provincial Medical Science and Technology Research Joint Venture Project (LHGJ20240154).

## Ethics Statement

The authors have nothing to report.

## Conflicts of Interest

The authors declare no conflicts of interest.

## Supporting information


**Figures S1–S9:** acel70332‐sup‐0001‐Figures.pdf.

**Table S1:** acel70332‐sup‐0002‐Table S1.xlsx.

**Table S2:** acel70332‐sup‐0003‐Table S2.xlsx.

## Data Availability

The authors affirm that all data substantiating the results of this investigation are accessible within the article and its Supporting Information files. All data will be shared upon reasonable request to the corresponding author. The vectors employed in this study will be furnished by the corresponding author upon reasonable request.
