# Supplementary material for: YTHDC1 Orchestrates Telomerase Assembly via Scaffold‐Mediated TERT‐TERC Interaction
Source: Aging Cell. 2025 Dec 28;25(1):e70332. doi: 10.1111/acel.70332 (PMC12745060; doi:10.1111/acel.70332)
Supplement: Supplementary file 1 — Figures S1–S9: acel70332‐sup‐0001‐Figures.pdf. [file ACEL-25-e70332-s003.pdf]

SUPPLEMENTARY INFORMATION

YTHDC1 Orchestrates Telomerase Assembly via Scaffold-Mediated TERT-TERC Interaction

Xiaolei Cheng[#1,2], Shixing Wang[#1], Yanan Yu[#1], Jianhang Xu[1], Qian Wang[1], Yuzhu Wei[3], Zeming Jin[4], Xinkun Qi[1], Dongdong Jian[1], Yingchao Shi[1], Zhen Li[1], Zhengliang Ma[2], Wengong Wang[3], Tianjiao Xia[5], Junyue Xing[*1], Xiaoping Gu[*2], Hao Tang[*1,4]

Figure S1

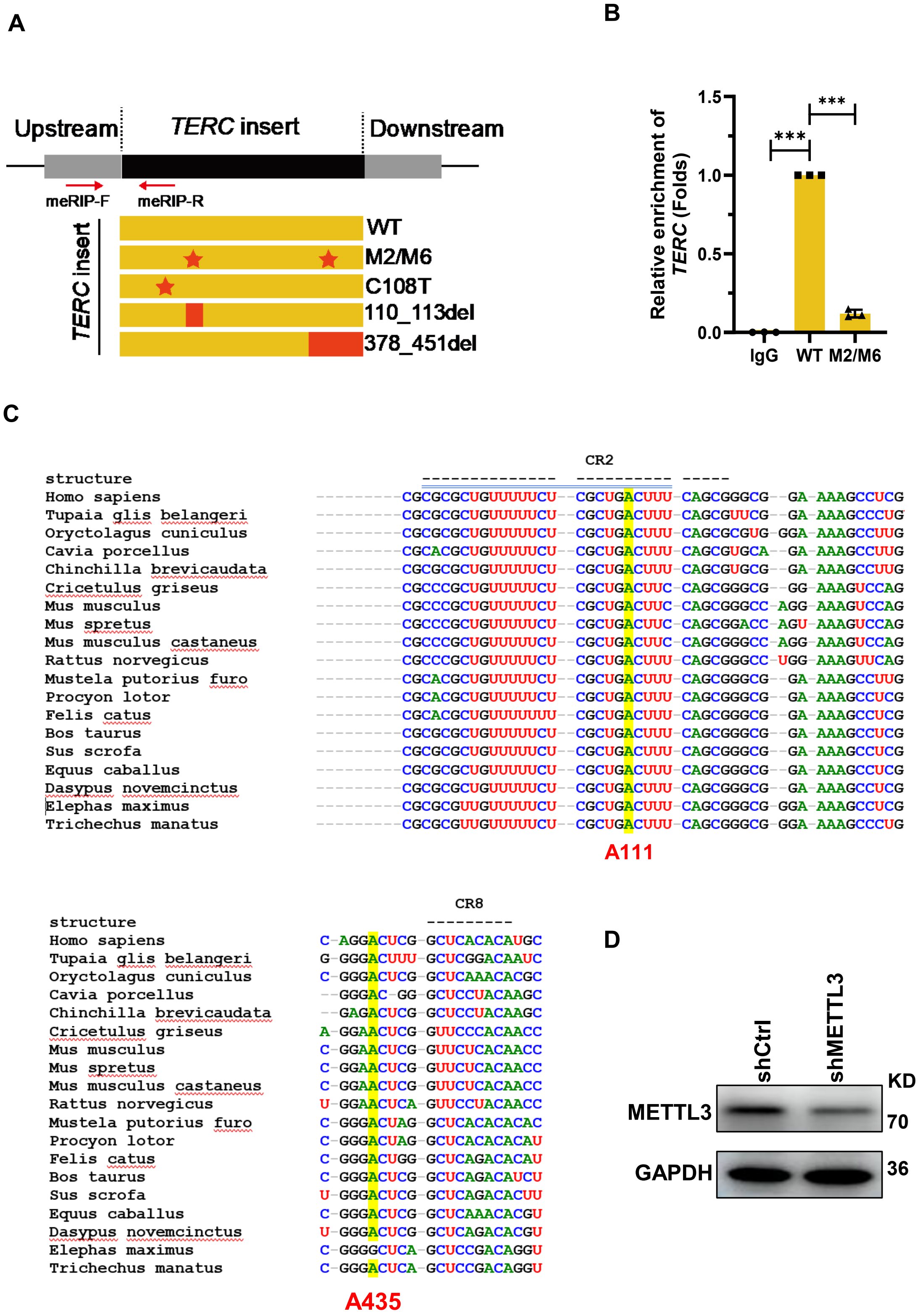

Figure S1 METTL3 mediates m6A methylation of *TERC* RNA. (A), Schematic of predicted m6A methylation sites (red) and PF-associated mutations in the *TERC* transcript. Red arrows indicate primer pairs used for detecting exogenous *TERC* in MeRIP-qPCR. **(B)**, AECs were transfected with wild-type

(WT) or mutant *TERC* transcripts (M2/M6, A111G/A435G). Forty-eight hours later, total RNA was extracted and subjected to m6A RNA immunoprecipitation (RIP) to assess m6A methylation levels. Fold enrichment was normalized to input and the WT group. ns, no significance. ***, $p<0.001$. (n=3). **(C)**, Evolutionary conservation analysis of *TERC* sequences across vertebrates. **(D)**, Western blot analysis of METTL3 expression in cells stably expressing the shMETTL3 virus.

Figure S2

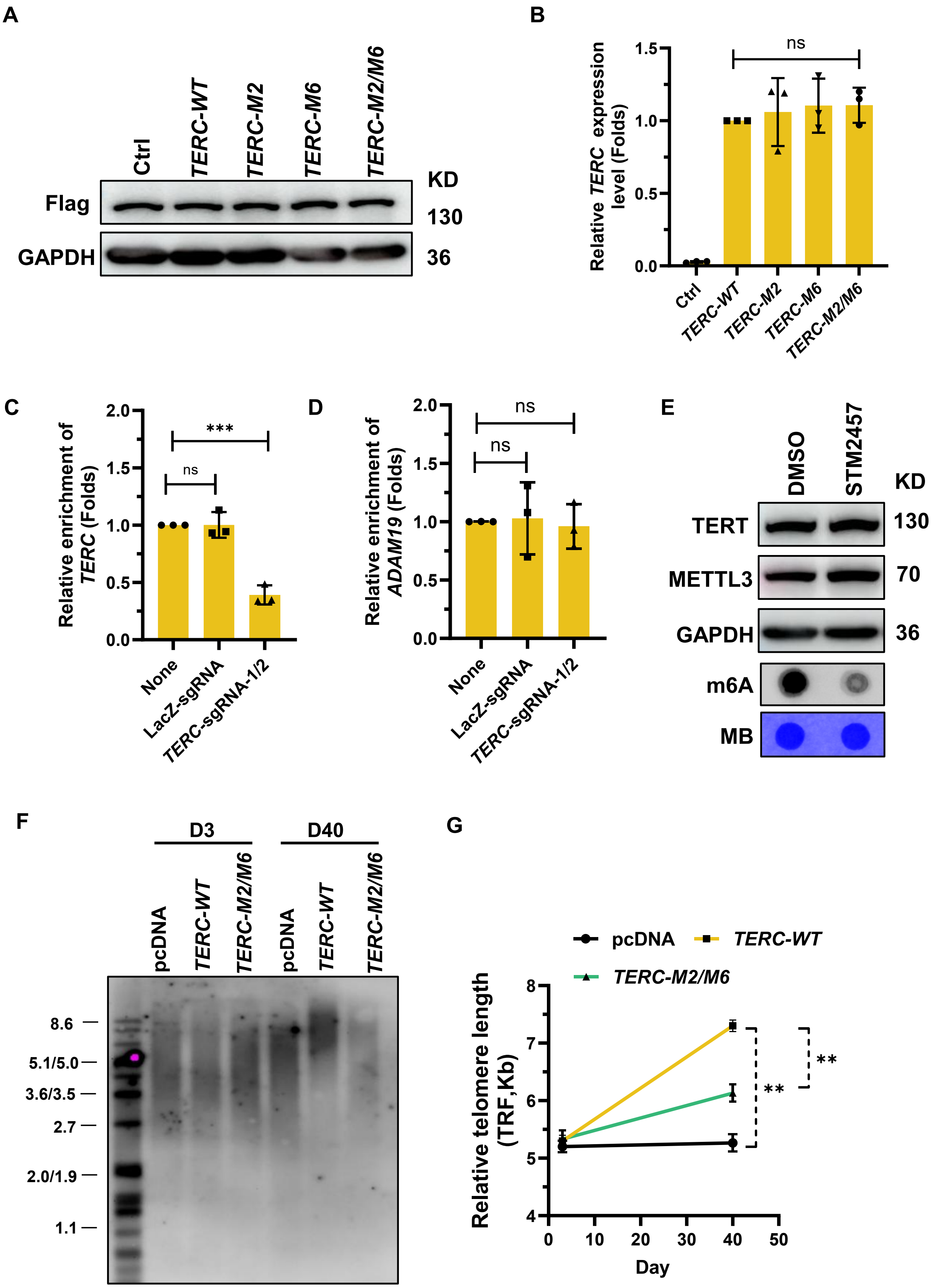
A
Ctrl
TERC-WT
TERC-M2
TERC-M6
TERC-M2/M6
KD
Flag
130
GAPDH
36
B
Relative TERC expression level (Folds)
ns
1.5
1.0
0.5
0.0
Ctrl
TERC-WT
TERC-M2
TERC-M6
TERC-M2/M6
C
Relative enrichment of TERC (Folds)
2.0
1.5
1.0
0.5
0.0
***
ns
None
LacZ-sgRNA
TERC-sgRNA-1/2
D
Relative enrichment of ADAM19 (Folds)
2.0
1.5
1.0
0.5
0.0
ns
ns
None
LacZ-sgRNA
TERC-sgRNA-1/2
E
DMSO
STM2457
KD
TERT
130
METTL3
70
GAPDH
36
m6A
MB
F
D3
D40
pcDNA
TERC-WT
TERC-M2/M6
pcDNA
TERC-WT
TERC-M2/M6
8.6
5.1/5.0
3.6/3.5
2.7
2.0/1.9
1.1
G
pcDNA
TERC-WT
TERC-M2/M6
Relative telomere length (TRF,Kb)
8
7
6
5
4
**
**
0
10
20
30
40
50
Day

Figure S2 m6A-modified *TERC* promotes telomerase activity and telomere maintenance. (A-B), Cells described in Figure 2C were subjected to Western blotting to evaluate Flag-TERT protein levels (A) or harvested for RNA extraction and RT-qPCR to assess the *TERC* expression level (B). **(C-D)**, MeRIP assessment of m6A methylation on *TERC* RNA and *ADAM19* RNA following TERC-targeted demethylation using the dCasRx-ALKBH5 editing system. **(E)** AECs treated with DMSO or STM2457 were subjected to Western blotting and RNA m6A dot blot assay. **(F-G)**, AECs stably expressing Flag-TERT were transfected with control, wild-type *TERC*, or mutant *TERC* transcripts (M2/M6, A111G/A435G) every three days. The cells were collected at indicated time points and subjected to southern blot to measure the average telomere length. **, p<0.01.(n=3).

25 **Figure S3**

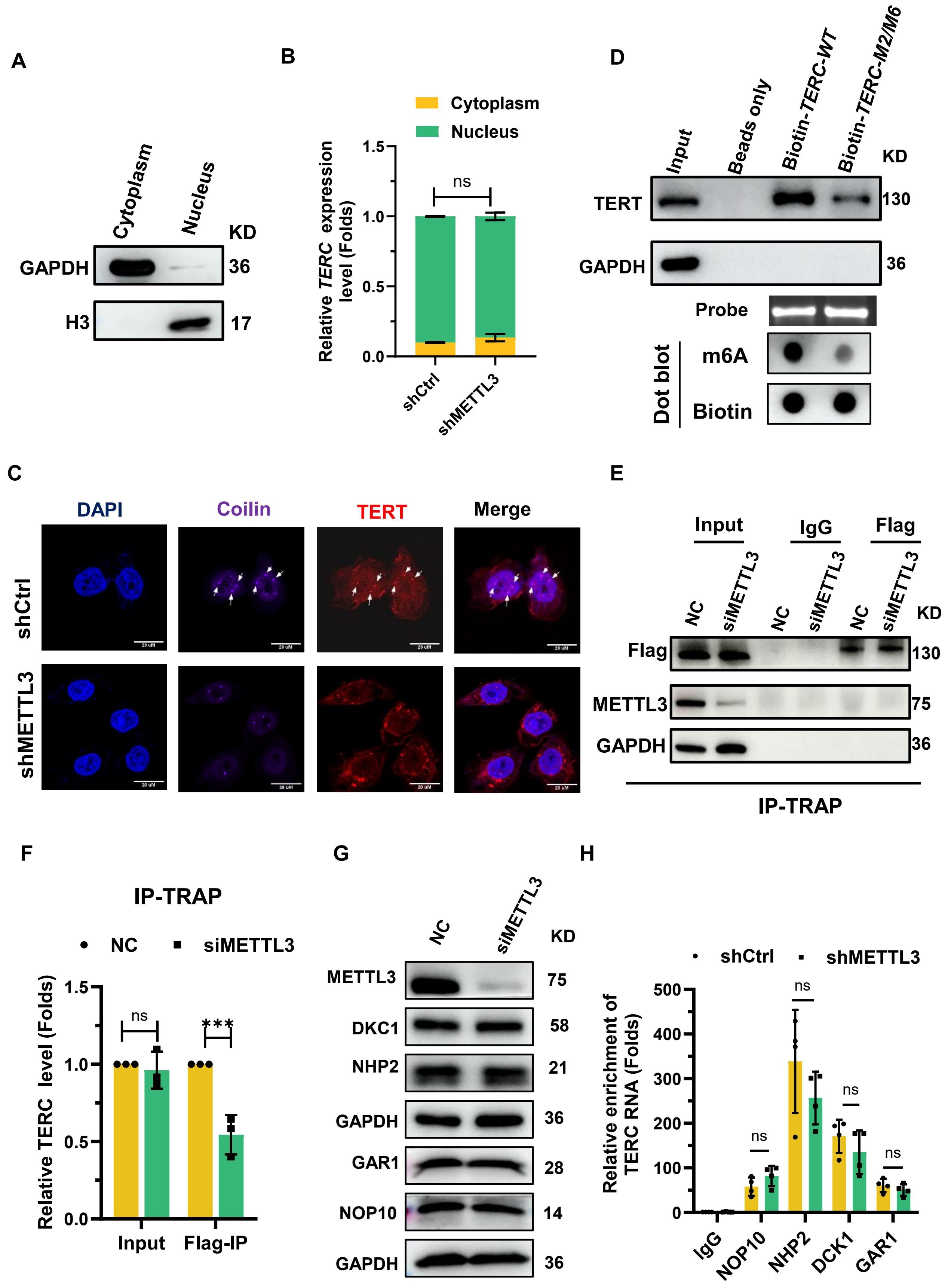

26

Figure S3 m6A-modified *TERC* strengthens TERT-*TERC* assembly. (A-B), RNA was isolated from separated cytoplasmic and nuclear fractions, and *TERC* expression levels in each compartment were quantified by RT-qPCR. **(C)**, Representative immunofluorescence imaging of AECs showing TERT (red), coilin (Cajal body marker, purple), and DAPI (nuclear stain, blue) in control (shCtrl) and METTL3-knockdown (shMETTL3) cells. Scale bar: 20 μm. **(D)**, AECs were subjected to RNA pulldown with biotin-labelled wild type or double mutant *TERC* probe (M2/M6, A111G/A435G). Top: Western blotting of TERT in pulldown material; Middle: Loading control; Bottom: RNA dot blot confirming m6A levels in probes. **(E-F),** Western blot and RT-qPCR assays were carried out to examine the Flag-TERT or TERC expression level in IP-TRAP materials. ***， $p<0.001$.(n=3). **(G)**, Western blotting was performed to assess the expression levels of *TERC*-associated proteins (DKC1, NOP10, GAR1, and NHP2). **(H),** RIP-qPCR was conducted to evaluate the interaction between *TERC*-associated proteins and *TERC.* ns, no significance. (n=4).

Figure S4

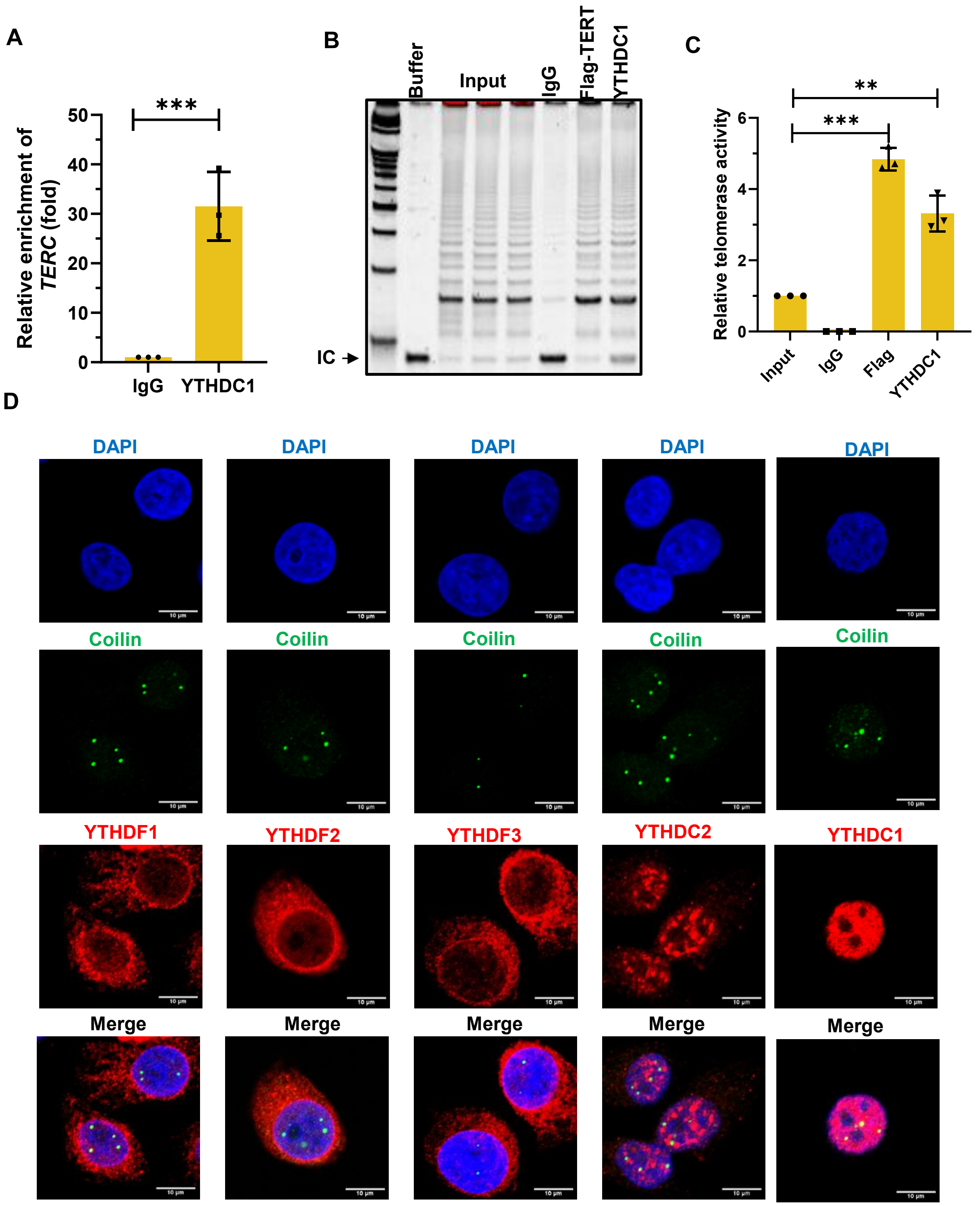

Figure S4 YTHDC1 binds to m6A-modified *TERC*. (A), AECs were exposed to UV crosslinking followed by RNA IP with YTHDC1 antibody to analyze the combination of YTHDC1 with *TERC*. ***, *p*<0.001. (n=3). **(B-C)**, AECs were subjected to IP assay using indicated antibodies. The IP materials were used to assess the telomerase activity with TRAP assays. IgG was used as negative control and

Flag-TERT was used as positive control. **, $p<0.01$; ***, $p<0.001$. (n=3). **(D)**, Representative immunofluorescence image of coilin (green) and YTH domain family protein (red). Scale bar, 10 μm.

Figure S5

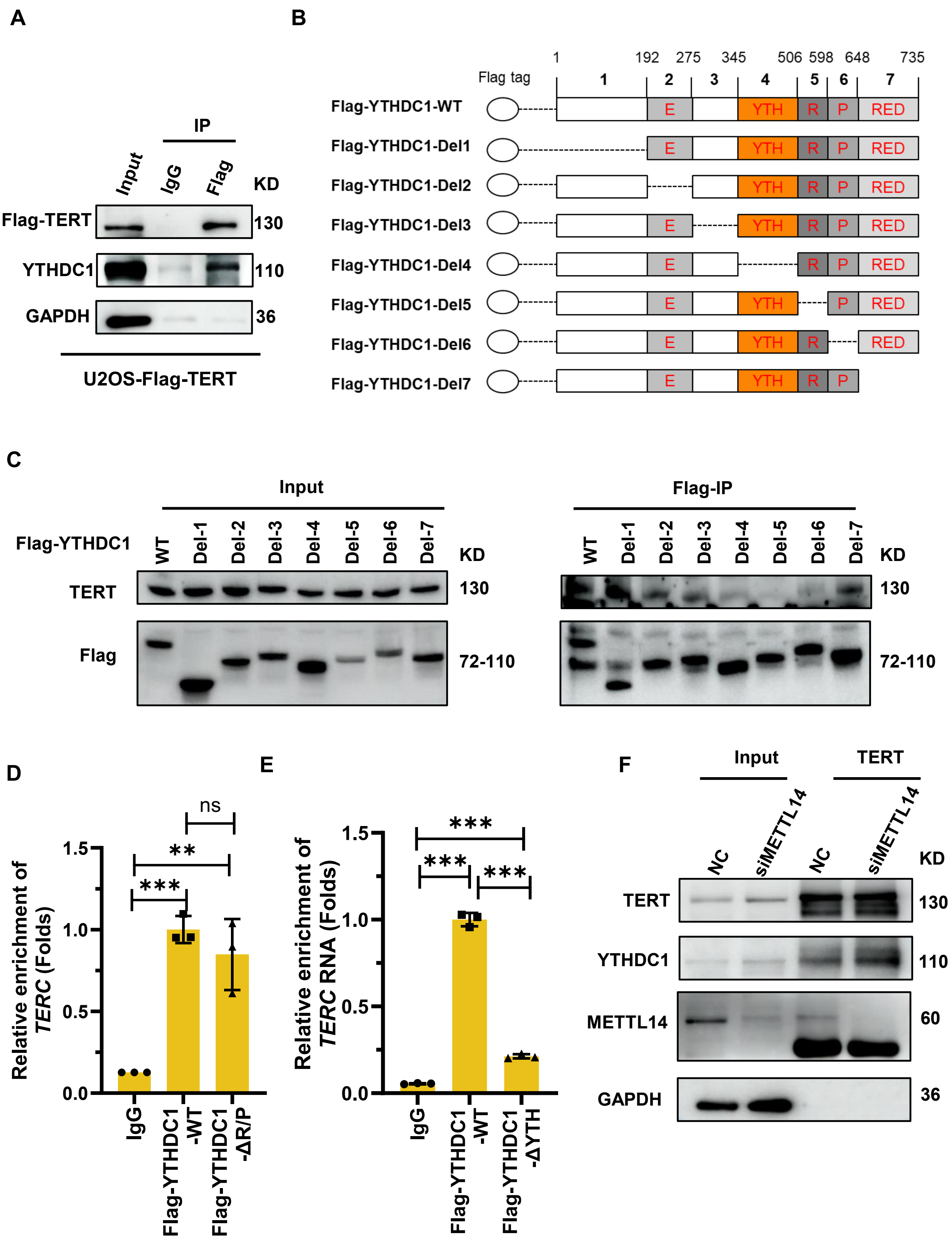

Figure S5 YTHDC1 binds directly with TERT. (A), U2OS cells were transfected with plasmid expressing Flag-TERT and subsequently subjected to co-immunoprecipitation using a Flag antibody to

assess the association of TERT with YTHDC1 in the absence of *TERC*. **(B),** Schematic representation of Flag-tagged YTHDC1 domain deletion constructs used in this study. **(C)**, AECs transfected with plasmids expressing Flag-YTHDC1-WT, del-1, del-2, del-3, del-4, del-5, del-6 or del-7 were subjected to immunoprecipitation using a Flag antibody, and the IP materials were analyzed by western blotting for indicated proteins. **(D)**, The cells described in Figure 5E were subjected to UV-crosslinking following by RIP with a Flag antibody to analyze the association of YTHDC1 with *TERC*. **, p<0.01; ***, p<0.001.(n=3). **(E)**, RIP assay was performed to evaluate the interaction between YTHDC1-del4 (YTH domain deletion) mutant and *TERC*. ***, p<0.001.(n=3). **(F)**, AECs were transfected with control (NC) or METTL14 siRNA (siMETTL14). Co-IP was performed using a TERT antibody, followed by Western blot analysis for YTHDC1.

Figure S6

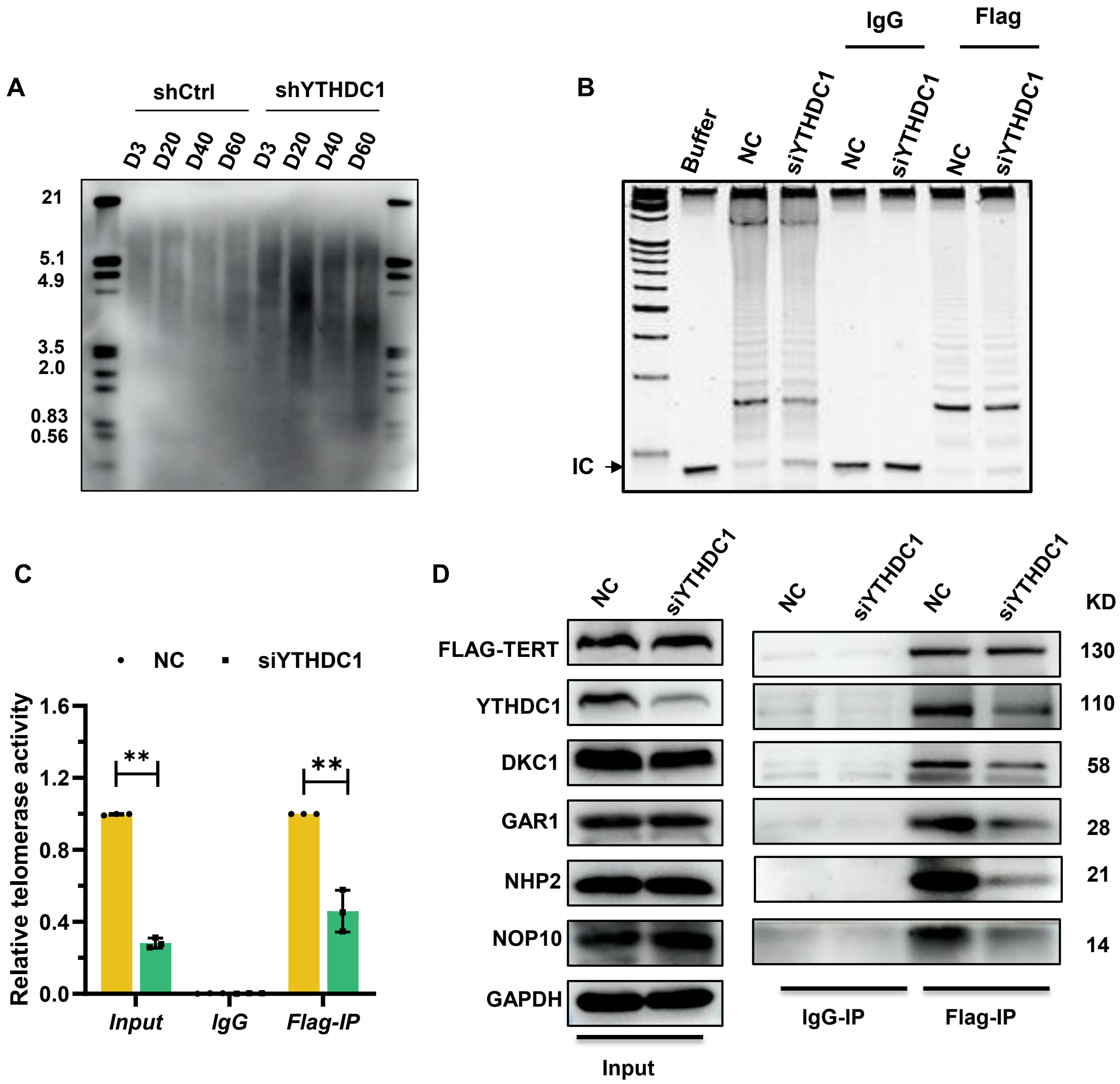

Figure S6 YTHDC1 enhances the telomerase activity and maintains the telomere length. (A), Southern blot analysis of telomere length in AECs stably expressing control shRNA (shCtrl) or YTHDC1-targeting shRNA (shYTHDC1) over serial passages. **(B-C)**, AECs stably expressing Flag-TERT were transfected with control siRNA (NC) or YTHDC1 siRNA (siYTHDC1). Forty-eight hours later, telomerase complexes were immunoprecipitated using a Flag antibody, and the IP materials were subjected to the TRAP assay. **, p<0.01.(n=3). **(D)**, The lysates from the cells described in Figure S6B were immunoprecipitated with a Flag antibody. The IP materials were analyzed by western blotting for the TERC-associated proteins.

Figure S7

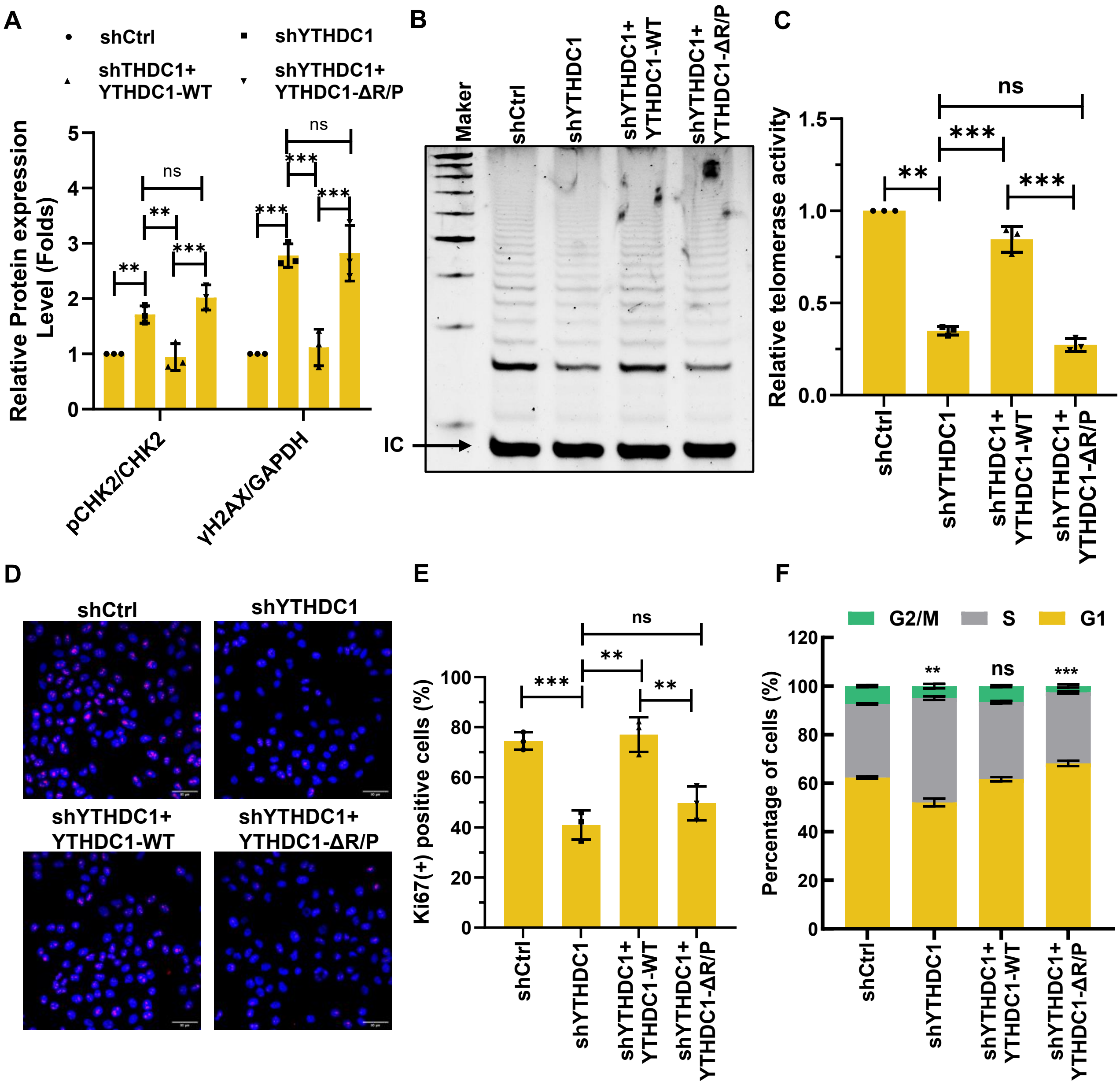

Figure S7 YTHDC1-mediated telomerase assembly enhances proliferation and antagonizes senescence in AEC cells. (A), Quantification of the γ-H2AX and p-CHK2 protein levels from western blotting analysis in Figure 6A. **, *p*<0.01; ***, *p*<0.001. (n=3). **(B-C),** Telomerase activity was measured by TRAP assay in cells described in Figure 6. **, *p*<0.01.(n=3). **(D-E)**, Cells from Figure 6 were subjected to ki67 immunofluorescence to evaluate the cell proliferation. **, *p*<0.01; ***, *p*<0.001. (n=3). **(F)**, Cell cycle analysis by flow cytometry in cells described in Figure 6. **, *p*<0.01. ***, *p*<0.001. (n=3).

Figure S8

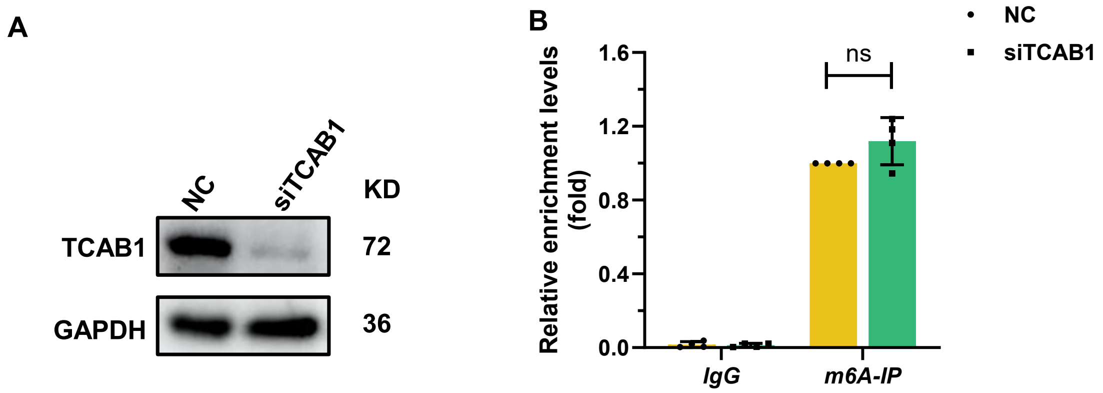

Figure S8 TCAB1 has no effect on m6A modification of *TERC*. (A), Western blotting analysis of TCAB1 knockdown efficiency in AECs. (B), m6A methylated RNA immunoprecipitation (MeRIP) assay using anti-m6A antibody or IgG control in TCAB1-depleted cells. ns, no significance. (n=4).

Figure S9

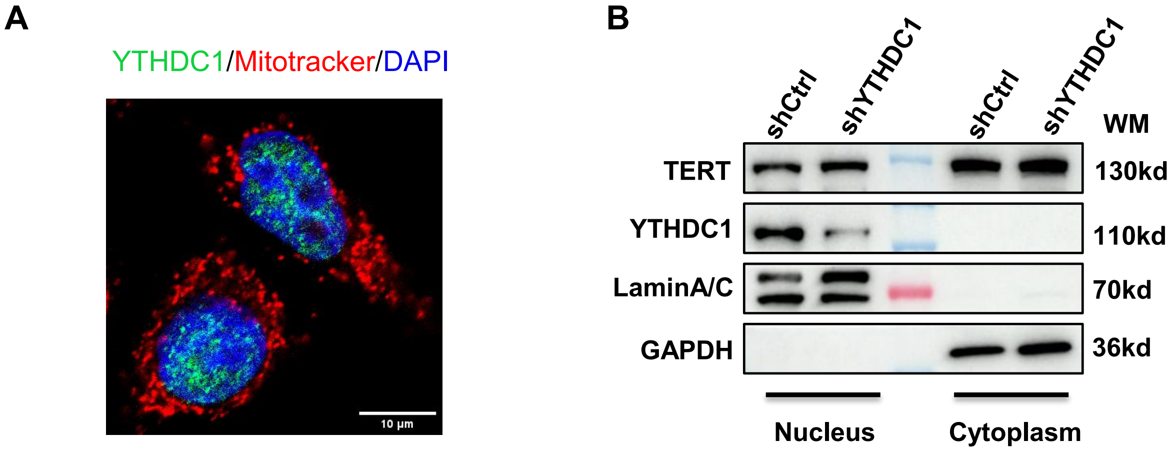

Figure S9 YTHDC1 knockdown does not alter the subcellular localization of TERT. (A), Immunofluorescence staining of YTHDC1 (green) in cells labeled with MitoTracker (red) and DAPI (blue). Scale bar: 10 µm. **(B),** Cytoplasmic and nuclear fractions were separated and analyzed by Western blotting to assess TERT distribution following YTHDC1 knockdown.
